# Epidemiologic and Genetic Associations of Endometriosis With Depression, Anxiety, and Eating Disorders

**DOI:** 10.1001/jamanetworkopen.2022.51214

**Published:** 2023-01-18

**Authors:** Dora Koller, Gita A. Pathak, Frank R. Wendt, Daniel S. Tylee, Daniel F. Levey, Cassie Overstreet, Joel Gelernter, Hugh S. Taylor, Renato Polimanti

**Affiliations:** 1 Department of Psychiatry, Yale School of Medicine, West Haven, Connecticut; 2 Veterans Affairs Connecticut Healthcare Center, West Haven; 3 Department of Genetics, Microbiology, and Statistics, Faculty of Biology, University of Barcelona, Catalonia, Spain; 4 Department of Obstetrics, Gynecology, and Reproductive Sciences, Yale School of Medicine, New Haven, Connecticut

## Abstract

**Importance:**

Endometriosis is a common chronic gynecologic pathology with a large negative impact on women’s health. Beyond severe physical symptoms, endometriosis is also associated with several psychiatric comorbidities, including depression and anxiety.

**Objective:**

To investigate whether pleiotropy contributes to the association of endometriosis with depression, anxiety, and eating disorders.

**Design, Setting, and Participants:**

This genetic association study was performed between September 13, 2021, and June 24, 2022, in 202 276 unrelated female participants. Genotypic and phenotypic information from the UK Biobank was combined with genome-wide association statistics available from the Psychiatric Genomics Consortium (11 countries), the Million Veteran Program (US), the FinnGen study (Finland), and the CHARGE (Cohorts for Heart and Aging Research in Genomic Epidemiology) consortium (5 countries).

**Main Outcomes and Measures:**

The main outcomes were the phenotypic and genetic associations of endometriosis with anxiety, depression, and eating disorders.

**Results:**

A total of 8276 women with endometriosis (mean [SD] age, 53.1 [7.9] years) and 194 000 female controls (mean [SD] age, 56.7 [7.9] years) were included in the study. In a multivariate regression analysis accounting for age, body mass index, socioeconomic status, chronic pain–related phenotypes, irritable bowel syndrome, and psychiatric comorbidities, endometriosis was associated with increased odds of depression (odds ratio [OR], 3.61; 95% CI, 3.32-3.92), eating disorders (OR, 2.94; 95% CI, 1.96-4.41), and anxiety (OR, 2.61; 95% CI, 2.30-2.97). These associations were supported by consistent genetic correlations (rg) (depression rg, 0.36, *P* = 1.5 × 10^−9^; anxiety rg, 0.33, *P* = 1.17 × 10^−5^; and eating disorders rg, 0.61, *P* = .02). With the application of a 1-sample mendelian randomization, the genetic liabilities to depression and anxiety were associated with increased odds of endometriosis (depression: OR, 1.09; 95% CI, 1.08-1.11; anxiety: OR, 1.39; 95% CI, 1.13-1.65). A genome-wide analysis of pleiotropic associations shared between endometriosis and psychiatric disorders identified 1 locus, *DGKB* rs12666606, with evidence of pleiotropy between endometriosis and depression after multiple testing correction (*z* = −9.46 for endometriosis, *z* = 8.10 for depression, *P* = 5.56 × 10^−8^; false discovery rate q = 4.95 × 10^−4^).

**Conclusions and Relevance:**

These findings highlight that endometriosis is associated with women’s mental health through pleiotropic mechanisms. To our knowledge, this is the first large-scale study to provide genetic and phenotypic evidence of the processes underlying the psychiatric comorbidities of endometriosis.

## Key Points

**Question:**

What biological mechanisms are associated with endometriosis with mental health comorbidities?

**Findings:**

This genetic association study of 8276 women with endometriosis and 194 000 female controls found that pleiotropy is likely associated with the comorbid depression, anxiety, and eating disorders observed in patients with endometriosis.

**Meaning:**

This study highlights the importance of considering endometriosis pathogenesis from a more comprehensive perspective, including both mental and physical health.

## Teaser

This genetic association study uses phenotypic and genetic association analyses of unrelated women to test the comorbidity of endometriosis with depression, anxiety, and eating disorders.

## Introduction

Endometriosis is a chronic disease characterized by endometriotic implants primarily on the pelvic peritoneum, ovaries, and rectovaginal septum,^1^ with 75% of patients experiencing chronic pelvic pain.^2^ Depression and anxiety are more prevalent among patients with endometriosis compared with the general population.^3^ Comorbid depression and anxiety have been associated with worse endometriosis symptoms, poor prognosis, and lower quality of life.^4,5^ The association between endometriosis and depression and anxiety has been previously linked to chronic pain.^3,6^ Although chronic pain surely plays an important role underlying these associations, it is not the sole factor. Animal models of endometriosis demonstrated that depression and anxiety are caused by endometriosis independent of pain,^7^ and women with endometriosis present a lower mean body weight.^8^

**Figure 1.**
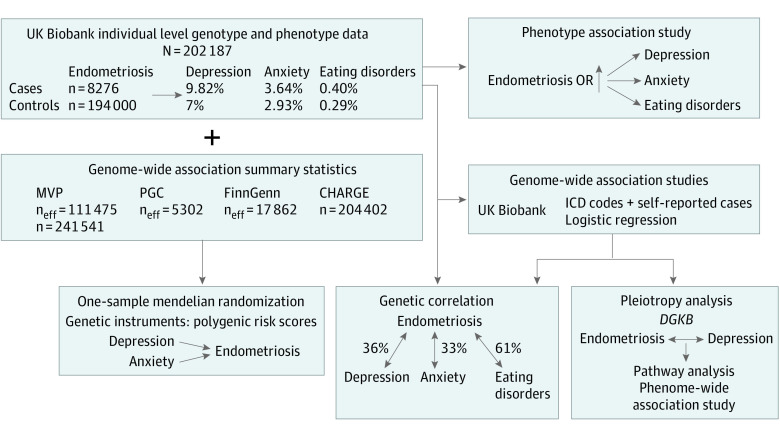
Study Overview A logistic regression analysis was performed to investigate whether psychiatric phenotypes are associated with endometriosis after accounting for multiple variables. Genetic correlation, mendelian randomization, and pleiotropy analyses were performed to study the genetic overlap between endometriosis and psychiatric traits. CHARGE indicates Cohorts for Heart and Aging Research in Genomic Epidemiology; MVP, Million Veteran Program; and PGC, Psychiatric Genomics Consortium.

To our knowledge, only 1 study has investigated the comorbidity between endometriosis and depression using genome-wide data,^9^ reporting a genetic correlation (rg) between these traits (rg = 0.27) and suggesting a potential causal effect of depression on endometriosis.^9^ Further analyses are needed to investigate the comorbidities of endometriosis across the psychopathology spectrum. In addition, it is important to understand whether the pleiotropy observed is due to cause-effect relationships or to shared biological pathways. To test these hypotheses, we combine individual-level data from the UK Biobank (UKB) with genome-wide association statistics from large collaborative studies, including Psychiatric Genomics Consortium (PGC) (11 countries), the Million Veteran Program (MVP) (US), the FinnGen study (Finland), and the CHARGE (Cohorts for Heart and Aging Research in Genomic Epidemiology) consortium (5 countries) (Figure 1), to conduct phenotypic and genetic association analyses testing the comorbidity of endometriosis with depression, anxiety, and eating disorders.

## Methods

A schematic workflow summarizing the analyses conducted is reported in Figure 1. These analyses were conducted from September 13, 2021, to June 24, 2022, in 202 276 unrelated female participants. The UKB has approval from the Northwest Multi-center Research Ethics Committee as a Research Tissue Bank (RTB). This approval means that researchers do not require separate ethical clearance and can operate under the RTB approval. The current study was conducted under an approved project. The PGC, MVP, FinnGen, and CHARGE genome-wide association statistics are previously collected, deidentified, aggregated data, and their analysis in the current study did not qualify as human participant research according to the 2018 Revised Common Rule of the Office of Human Research Protections. The study was reported in accordance with the Strengthening the Reporting of Genetic Association Studies (STREGA) reporting guideline.^10^

### UK Biobank Cohort: Self-reported and *ICD-10* Definitions

We used individual-level genotype and phenotype data from unrelated female participants of European descent (Table 1) available from the UKB.^11^ Because of the limited sample size available, we were not able to analyze other ancestry groups.

**Table 1.** Characteristics of the UK Biobank Sample Investigated in the Current Study^a^

Characteristic	Cases (n = 8276)	Controls (n = 194 000)	*P* value
Age at recruitment, mean (SD), y	53.1 (7.9)	56.7 (7.9)	<.001
Depression	813 (9.8)	13 584 (7.)0	<.001
Anxiety	301 (3.6)	5688 (2.9)	<.001
Eating disorders	33 (0.4)	558 (0.3)	<.001
BMI, mean (SD)	27.61 (5.39)	26.98 (5.14)	<.001
Townsend Deprivation Index at recruitment, mean (SD)^b^	−1.28 (3.00)	−1.56 (2.91)	<.001
Age at menarche, mean (SD), y	12.8 (1.7)	13.0 (1.6)	<.001
Ever taken oral contraceptive pill	7065 (85.4)	155 164 (80.0)	<.001
Back pain for ≥3 mo	2002 (24.2)	32 461 (16.7)	<.001
Stomach or abdominal pain for ≥3 mo	1044 (12.6)	9506 (4.9)	<.001
Medication for pain relief, constipation, or heartburn			
Aspirin	707 (8.5)	18 510 (9.5)	.002
Ibuprofen	1722 (20.8)	29 524 (15.2)	<.001
Paracetamol	1561 (18.9)	29 410 (15.2)	<.001
Ranitidine	61 (0.7)	1384 (0.7)	.75
Omeprazole	284 (3.4)	5315 (2.7)	<.001
Laxatives	143 (1.7)	2648 (1.4)	.005
None of the above	3656 (44.2)	100 073 (51.6)	<.001
Pain type(s) experienced in last month			
Headache	2740 (33.1)	43 601 (22.5)	<.001
Facial pain	97 (1.2)	1610 (0.8)	<.001
Neck or shoulder pain	1240 (15.0)	26 416 (13.6)	<.001
Back pain	827 (10.0)	19 463 (10.0)	.91
Stomach or abdominal pain	317 (3.8)	4177 (2.2)	<.001
Hip pain	195 (2.4)	5786 (3.0)	.001
Knee pain	334 (4.0)	9662 (5.0)	<.001
Pain all over the body	224 (2.7)	3270 (1.7)	<.001
None of the above	2276 (27.5)	74 314 (38.3)	<.001
Ever diagnosed with IBS	763 (9.2)	11 182 (5.8)	<.001
Frequency of discomfort or pain in abdomen in last 3 mo (0 indicating never to 6 indicating every day), median (IQR)	2 (0-4)	1 (0-3)	<.001
Discomfort or pain occurring only during menstrual bleed	47 (0.6)	764 (0.4)	.01
Abdominal discomfort/pain for ≥6 mo	1405 (17.0)	24 191 (12.5)	<.001
Frequency of discomfort or pain getting better or stopping after a bowel movement (0 indicating never to 4 indicating always), median (IQR)	2 (1-3)	2 (1-3)	>.99
More frequent bowel movements when abdominal discomfort or pain started (0 indicating never to 4 indicating always), median (IQR)	1 (0-2)	1 (0-1)	>.99
Severity of current abdominal pain (0 indicating no pain to 10 indicating very severe pain), median (IQR)	5 (3-7)	4 (2-6)	<.001
No. of days (of 10) with abdominal pain (0 indicating no days with pain to 10 indicating 10 d with pain), mean (SD)	3.68 (2.83)	3.05 (2.59)	<.001
Length of menstrual cycle, mean (SD), d	26.69 (7.46)	26.85 (7.06)	.05

Abbreviations: BMI, body mass index (calculated as weight in kilograms divided by height in meters squared); IBS, irritable bowel syndrome.

^a^ Data are presented as number (percentage) of study participants unless otherwise indicated. The difference between cases and controls was evaluated using a 2-tailed, unpaired *t* test for continuous traits and a χ^2^ test for categorical traits.

^b^ Percentage of non–car ownership, percentage of non–home ownership, percentage of unemployment, and percentage of overcrowding. *z* scores = (percentage – mean of all percentages)/SD of all percentages for a total of the 4 *z* scores.

Ancestry assignment and relatedness assessment were performed as previously reported.^11^ To maximize the sample size for affected individuals, we combined self-reported information and diagnoses available from UKB electronic health records. We identified 8276 individuals with endometriosis: 4998 with *International Statistical Classification of Diseases and Related Health Problems, Tenth Revision* (*ICD-10*) codes in their electronic health records (*ICD-10* code N80) and 4300 with self-reported diagnoses (diagnosis 20002_1402) from the UKB surveys (1022 presented with both *ICD-10* codes and a self-reported diagnosis) (eMethods in Supplement 1). Although only 12% of patients with endometriosis had both *ICD-10* codes and self-reported diagnoses, we observed a complete genetic correlation between these 2 phenotypic definitions (rg = 1, *P* = 1.26 × 10^−7^). We defined 14 397 cases based on *ICD-10* codes and self-reported information for depression, 5989 for anxiety, and 591 for eating disorders (eMethods and eTable 1 in Supplement 1). The overlap between *ICD-10* codes and self-reported diagnoses were 67% for depression, 46% for anxiety, and 75% for eating disorders. In a previous study,^12^ consistent genetic effects were detected across different anxiety and depression case definitions available from the UKB.

### Logistic Regression: Phenotype Association

The phenotype association of endometriosis with anxiety, depression, and eating disorders was evaluated using logistic regression models with endometriosis as the main variable. The analysis was performed using the glm function of the stats package in R software, version 4.1.2 (R Foundation for Statistical Computing). Linearity, homoscedasticity, normality, and independence were evaluated via diagnostic plots drawn using the plot() function in R. The analysis was performed in UKB unrelated female participants (8276 women with endometriosis and 194 000 controls). Based on previous knowledge about their association with endometriosis,^13,14,15,16,17,18^ 4 models were defined using different sets of covariates (Table 2; eMethods in Supplement 1).

**Table 2.** Association of Endometriosis With Depression, Anxiety, and Eating Disorders Considering Different Sets of Covariates^a^

Model	Odds ratio (95% CI)		
	Depression	Anxiety	Eating disorders
1	4.54 (4.42-4.78)	3.35 (2.96-3.80)	5.02 (3.42-7.38)
2	4.14 (3.82-4.49)	3.16 (2.79-3.58)	4.91 (3.33-7.24)
3	3.65 (3.37-3.97)	2.70 (2.38-3.07)	4.05 (2.73-6.02)
4	3.61 (3.32-3.92)	2.61 (2.30-2.97)	2.94 (1.96-4.41)

^a^ Model 1 covariate was age. Model 2 covariates were age, body mass index, socioeconomic status, age at menarche, and length of menstrual cycle. Model 3 covariates were model 2 covariates plus ever taken oral contraceptive pill, medication for pain relief, constipation, heartburn, irritable bowel syndrome, and pain phenotypes. Model 4 covariates were model 3 covariates plus other psychiatric diagnoses. All associations reported survived Bonferroni multiple testing correction.

### Genome-Wide Association Study

Female-only genome-wide association studies (GWASs) were generated based on the phenotype definitions described above. For anxiety, depression, and eating disorders, we conducted a genome-wide association analysis for each of them according to the phenotype definition based on *ICD-10* codes and self-reported information. The analyses were conducted with PLINK, version 1.9^19^ using a logistic regression model and covarying for age and the first 10 within-ancestry principal components. We also used GWAS data from previous large-scale studies^20,21,22^ to investigate further the genetic associations of endometriosis with depression, anxiety, and eating disorders (eMethods in Supplement 1).

### Heritability and Genetic Correlation Based on Single-Nucleotide Variants

We applied the Scalable Genetic Correlation Estimator (SCORE)^23^ method to compute single-nucleotide variant (SNV)–based heritability and genetic correlation from individual genotype and phenotype data with the addition of covariates (age, first 10 within-ancestry principal components, and the other psychiatric traits [as described above]). We also applied linkage disequilibrium score regression (LDSC)^24^ to calculate SNV-based heritability and genetic correlation using genome-wide association statistics generated from UKB and those obtained from previous studies^20,21,22,25^ (eMethods in Supplement 1).

### One-Sample Mendelian Randomization

Bidirectional 1-sample mendelian randomization was performed to test the causal associations among endometriosis, anxiety, depression, and eating disorders. The instrumental-variable regression (ivreg) function of the Applied Econometrics package in R^26^ was used to perform 2-stage least squares estimation. Exposures were instrumented by creating a polygenic risk score (PRS). The PRSs were calculated using PRSice-2 software, version 2.3.3^27^ (eMethods in Supplement 1). All exposures and outcomes were binary variables. Effect estimates were generated from the 2-stage least squares regression. Sensitivity and power analyses were also performed (eMethods in Supplement 1).

### Pleiotropy Analysis

To investigate pleiotropic loci associated with endometriosis, depression, anxiety, and eating disorders, we used the PolarMorphism R package, version 1.^28^ Variants with a nominally significant pleiotropy were used to perform a gene-based analysis with the Versatile Gene-based Association Study 2 tool.^29^ The test uses information from a set of markers while accounting for linkage disequilibrium. Based on the gene-based associations, an enrichment analysis was performed with respect to gene sets from the gene ontology, biological pathways, and molecular functions.^29^ To characterize further the rs12666606 pleiotropic variant, we performed a phenome-wide association study using female-specific data from UKB and sex-combined data from the GWAS Atlas^30^ (eMethods and eResults in Supplement 1).

## Results

### Phenotypic Associations

A total of 8276 women with endometriosis (mean [SD] age, 53.1 [7.9] years) and 194 000 female controls (mean [SD] age, 56.7 [7.9] years) were included in the study. To investigate differences due to sociodemographic characteristics and possible endometriosis symptoms, we tested multiple regression models accounting for different sets of covariates (Table 2; eTable 2 in Supplement 1). Accounting only for age (model 1), we observed that endometriosis was associated with increased odds of eating disorders (OR, 5.02; 95% CI, 3.42-7.38), depression (OR, 4.54; 95% CI, 4.42-4.78), and anxiety (OR, 3.35; 95% CI, 2.96-3.80). These associations remained significant after accounting for age, body mass index, Townsend Deprivation Index at recruitment, menarche, and length of menstrual cycle (model 2). We observed statistically significant changes in the effect estimates after adding possible endometriosis symptoms (ie, pain-related phenotypes and irritable bowel syndrome) to the previous covariates (model 3) for depression and anxiety but not for eating disorders (model 2 vs model 3: depression: *P* = 3.77 × 10^−3^; anxiety: *P* = .02; and eating disorders: *P* = .24). When psychiatric comorbidities were included with the previous covariates (eg, when investigating whether anxiety is associated with endometriosis, depression and eating disorders were added as covariates; model 4), endometriosis was associated with increased odds of negative mental health outcomes, with the largest association observed for depression (OR, 3.61; 95% CI, 3.32-3.92), followed by eating disorders (OR, 2.94; 95% CI, 1.96-4.41) and anxiety (OR, 2.61; 95% CI, 2.30-2.97). When comparing effect sizes between models 1 and 4 for all phenotypes, we observed statistically significant difference between the effect estimates (model 1 vs model 4: *P* = 7.59 × 10^−5^ for depression, *P* = 1.65 × 10^−4^ for anxiety, and *P* = 7.65 × 10^−3^ for eating disorders) (eTable 2 in Supplement 1). No violation of modeling assumptions was detected in the residual vs fitted, normal Q-Q scale location and residual vs leverage plots.

### SNV-Based Heritability and Genetic Correlation

**Figure 2.**
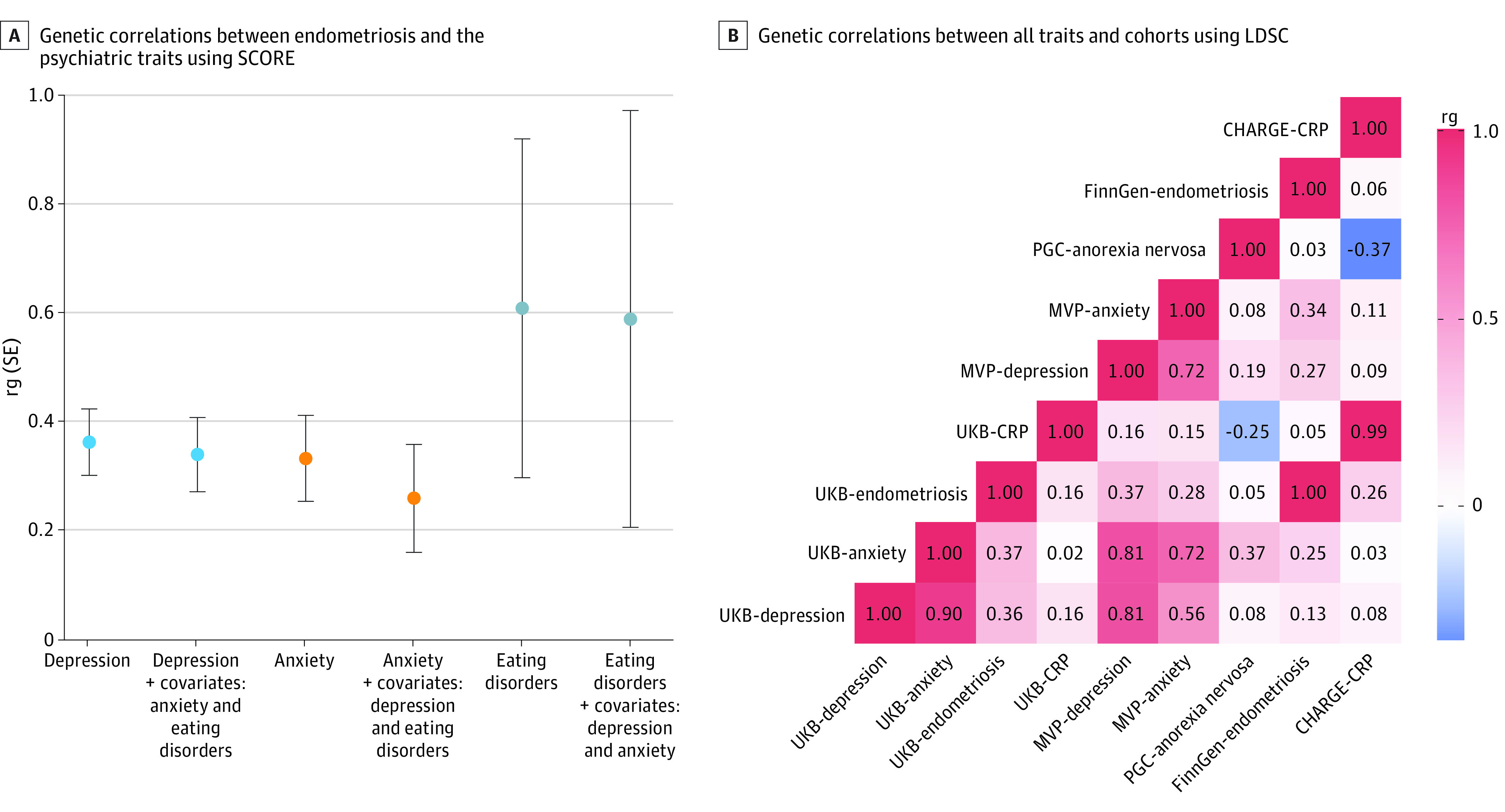
Genetic Correlation Among Traits and Cohorts Investigated in the Study A, Genetic correlation between endometriosis and depression, anxiety, and eating disorders using the Scalable Genetic Correlation Estimator (SCORE) with UK Biobank (UKB) individual-level genotype data with age and the first 10 principal components as covariates and additionally adding the other 2 traits as covariates. Whiskers represent standard errors. B, Genetic correlation across all traits and cohorts using linkage disequilibrium score regression (LDSC) with genome-wide association study summary statistics. CHARGE indicates Cohorts for Heart and Aging Research in Genomic Epidemiology; CRP, C-reactive protein; MVP, Million Veteran Program; PGC, Psychiatric Genomics Consortium; and rg, genetic correlation.

Applying the SCORE method to the UKB individual-level phenotypic and genetic data, we estimated female-specific SNV-based heritability (SNV- h^2^) for endometriosis (mean [SE] SNV-h^2^, 0.086 [0.015]), depression (SNV-h^2^, 0.019 [0.003]), anxiety (SNV-h^2^, 0.012 [0.002]), and eating disorders (SNV-h^2^, 0.004 [0.002]). Six genome-wide significant loci were observed for endometriosis (eTable 3 in Supplement 1) but none for depression, anxiety, and eating disorders. Statistics of the GWASs from LDSC are given in eTable 4 in Supplement 1. In line with the phenotypic associations, endometriosis was genetically correlated with depression (rg = 0.36, *P* = 1.5 × 10^−9^), anxiety (rg = 0.33, *P* = 1.17 × 10^−5^), and eating disorders (rg = 0.61, *P* = .03). After accounting for psychiatric comorbidities (when investigating whether anxiety is genetically correlated with endometriosis, depression and eating disorders were added as covariates in the SCORE analysis), the genetic correlations of these psychiatric disorders with endometriosis were still present, although their strength was attenuated (anxiety: rg = 0.26, *P* = 4.1 × 10^−3^; depression: rg = 0.34, *P* = 1 × 10^−5^; eating disorders: rg = 0.59, *P* = .06) (Figure 2A). To expand the genetic correlation analysis, we analyzed genome-wide association statistics using the LDSC approach. In line with SCORE analysis, endometriosis showed a consistent genetic correlation with anxiety (rg = 0.36, *P* = 3 × 10^−4^) and depression (rg = 0.34, *P* = 1 × 10^−5^) when applying LDSC to UKB female-specific genome-wide association statistics. When considering the genome-wide association statistics generated from other cohorts (FinnGen, MVP, and PGC), we observed a similar pattern of genetic correlations (Figure 2B; eTable 5 in Supplement 1). Specifically, we observed consistent genetic correlations between our female-specific GWASs from the UKB and other both-sex combined GWASs (MVP-UKB depression = 0.81, MVP-UKB anxiety = 0.72, and CHARGE-UKB C-reactive protein [CRP] = 0.99) (Figure 2B; eTable 5 in Supplement 1).

### One-Sample Mendelian Randomization

**Figure 3.**
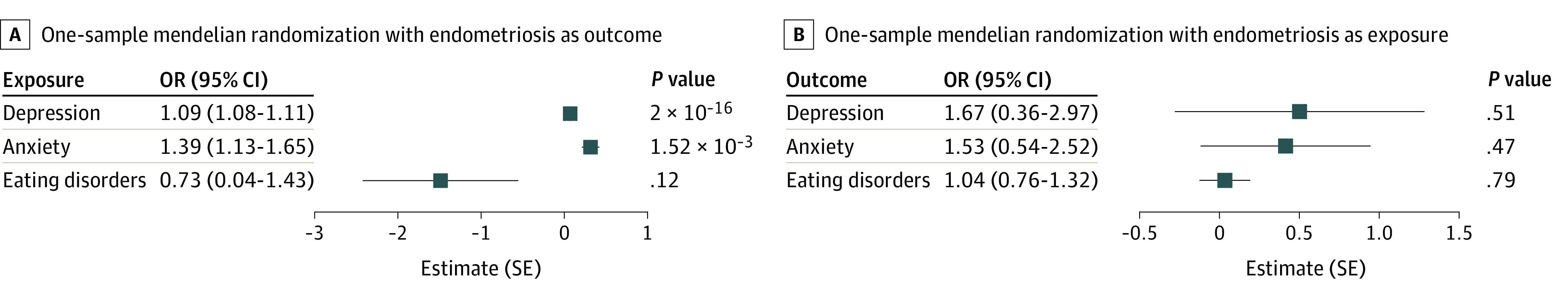
One-Sample Mendelian Randomization Analysis With Endometriosis as Outcome and Endometriosis as Exposure Bidirectional 1-sample mendelian randomization analyses were performed to examine the association between endometriosis and depression, anxiety, and eating disorders. OR indicates odds ratio.

The best-fit PRSs were used as genetic instruments in the 1-sample mendelian randomization analyses for each exposure (with *P* value thresholds of 0.05 for endometriosis, >.99 for depression, .05 for anxiety, >.99 for anorexia nervosa, and 10^−5^ for CRP) (eTable 6 in Supplement 1). Figure 3 shows the results of the mendelian randomization analysis. We found significant associations for depression (OR, 1.09; 95% CI, 1.08-1.11) and anxiety (OR, 1.39; 95% CI, 1.13-1.65) with endometriosis, but no association was observed with respect to eating disorders (OR, 0.73; 95% CI, 0.04-1.43); the latter null result was likely due to the lack of power of the GWAS of eating disorders as shown in our power analysis (power = 0.05) (eTable 7 in Supplement 1). Sensitivity analyses showed that there was no weak instrument bias (F>10; F = 7695, *P* < 2 × 10^−16^ for depression; F = 78.01, *P* < 2 × 10^−16^ for anxiety; and F = 11.15, *P* = 8.39 × 10^−4^ for eating disorder). The Wu-Hausman test showed that the instrumental variables are consistent, but the ordinary least squares were not; hence, the estimate with such a large sample size may not converge with the exact value (F = 10.39, *P* = 1.27 × 10^−3^ for depression and F = 120.5, *P* < 2 × 10^−16^ for anxiety). To investigate whether associations between psychiatric disorders and endometriosis may involve a shared inflammatory pathway, we conducted a 1-sample mendelian randomization analysis testing the association of CRP with endometriosis observing a null result (OR, 1.0; 95% CI, 0.998-1.00). Our power analysis revealed that the CRP GWAS was adequately powered (power = 1) (eTable 7 in Supplement 1). We also tested the possible association of endometriosis with the psychiatric disorders investigated, but the results were null due to a lack of power in the endometriosis GWAS. Indeed, our power analysis showed a lack of power in case of all outcomes (eTable 7 in Supplement 1).

### Pleiotropy Analysis

Only *DGKB* (OMIM 604070) rs12666606 pleiotropy between endometriosis and depression survived genome-wide multiple testing correction (*z* scores, −9.46 for endometriosis and 8.10 for depression; r = 12.45; θ *P* = 5.56 × 10^−8^, θ q = 4.95 × 10^−4^) (eTable 8 in Supplement 1). Performing an enrichment analysis of nominally significant gene-based associations (eTable 9 in Supplement 1), we observed that 3 pathways survived multiple testing correction (false discovery rate q <0.05) (eTable 10 in Supplement 1): diacylglycerol kinase activity, kinase (PANTHER Molecular Function), and activation of protein kinase C activity by G-protein–coupled receptor protein signaling pathway.

## Discussion

This genetic association study provides novel insights into the phenotypic and genetic associations underlying the psychiatric comorbidities of endometriosis. Our genetic and phenotypic analyses included a wide range of covariates to account for the possible effects of variables (eg, socioeconomic status, pain severity, and population stratification) that could affect the associations identified. Accordingly, our findings highlight that pleiotropy likely contributes to the adverse mental health outcomes observed in women affected by endometriosis independently of known risk factors and possible confounders.

When accounting for age, body mass index, socioeconomic status, age at menarche, length of menstrual cycle, irritable bowel syndrome, contraceptive medications, and several pain-related phenotypes, eating disorders were associated with higher odds of endometriosis than depression and anxiety. When we accounted for these factors together with psychiatric comorbidities, endometriosis was more strongly associated with depression than eating disorders and anxiety. Nevertheless, the phenotypic associations of endometriosis with the 3 psychiatric disorders investigated remained significant also after accounting for their comorbidity. Among the covariates tested, we observed that accounting for phenotypes associated with chronic pain reduced the odds of anxiety, depression, and eating disorders in women affected by endometriosis. Many patients with endometriosis experience constant pain regardless of their menstrual cycle phase.^31^ This severe chronic pain increases the risk of depression and other psychiatric comorbidities.^32^ In a clinical study, depression was detected in 86% of the patients with endometriosis and chronic pelvic pain compared with 38% of the patients without chronic pelvic pain.^33^ Although chronic pain is likely a key factor affecting these patients’ lives, including their mental health, quality of life, sexual health, social life, professional career, and other comorbidities,^5^ our results highlight that chronic pain does not completely explain the increased odds of anxiety, depression, and eating disorders observed in endometriosis cases. In mice, the effect of endometriosis on hippocampus, amygdala, and insula appears to cause pain sensitization and mood disorders.^7^

With respect to endometriosis case status, we observed a limited overlap (n = 1022 [12%]) between the self-reported diagnosis (n = 4300) and the *ICD-10*–based diagnosis (n = 4998). This finding is perhaps unsurprising because endometriosis is systemically underdiagnosed, and often diagnoses are delayed or altogether missed.^34^ However, a complete genetic correlation (rg = 1) was present between self-reported and *ICD-10*–based definitions within the UKB sample and between the combined UKB sample and the independent FinnGen cohort.^35^

To investigate endometriosis genetic correlations, we used 2 different methods (ie, LDSC and SCORE) that yielded comparable results for depression and anxiety (0.36 vs 0.36 for LDSC and 0.33 vs 0.37 for SCORE). Because of the limited statistical power of the eating disorder GWAS, a significant finding was detected only with the SCORE approach. Although the eating disorder GWAS has the lowest statistical power, we observed a stronger genetic correlation between endometriosis and eating disorders (rg = 0.61) than with anxiety (rg = 0.36) and depression (rg = 0.33). This pattern is in line with the strengths of observed phenotypic associations. When we reassessed the genetic correlations while controlling for effects of comorbid psychiatric conditions, the associations of endometriosis with depression and eating disorders appeared more robust, whereas the association with anxiety was reduced (from 0.33 to 0.26). However, similarly to phenotypic associations, genetic correlations remained statistically significant after accounting for the comorbidity among psychiatric disorders, suggesting there are disease-specific factors that contribute to the pleiotropy of endometriosis, with anxiety, depression, and eating disorders.

Our 1-sample mendelian randomization analyses found evidence of an association of depression and anxiety with endometriosis. The association of depression with endometriosis is in line with a previous 2-sample mendelian randomization study conducted on different cohorts.^9^ A phenotype-based longitudinal study^36^ based on a Swedish nationwide cohort found a bidirectional association of endometriosis with both depression and anxiety. Conversely, eating disorders appear to have a unidirectional association on endometriosis.^36^ Our study expands these previous findings, providing compelling genetic and phenotypic evidence of the dynamics underlying psychiatric comorbidities of endometriosis. The Wu-Hausman sensitivity test showed that the instrumental variables (eg, PRS for depression and anxiety) were consistent, but the ordinary least squares regressions were not. This phenomenon occurs when the estimates may be biased by the very large sample size.^37^ Because our findings are consistent with a previous study^9^ based on different methods and data, we hypothesize that this potential bias did not affect the estimates. Unfortunately, because of the lack of power of the GWAS data sets that were informative for endometriosis and eating disorders, we could not fully explore the possible association of endometriosis with psychiatric disorders and the association between endometriosis and eating disorders.

We also investigated the association of inflammation with endometriosis, with the goal of assessing the association observed for depression and anxiety. Elevated levels of CRP, the most commonly evaluated biomarker of acute systemic inflammation,^38^ were previously associated with endometriosis,^39^ depression,^40^ and anxiety.^41^ However, cross-sectional analysis of CRP levels cannot be useful to infer possible causal effects. Accordingly, we performed a genetically informed causal inference analysis based on CRP PRS. Nevertheless, our 1-sample mendelian randomization did not observe any effect of CRP PRS on endometriosis. Our power analysis confirmed that this null result was not due to a lack of power. A possible limitation of this analysis is that the CRP PRS was derived from a GWAS conducted in both women and men. Because of the strong differences in CRP levels between men and women,^42^ CRP genetic regulation could present major differences between sexes that affect our ability to investigate the association of CRP with endometriosis without an instrumental variable derived from sex-specific genetic associations. Furthermore, although women with endometriosis have greater odds of having elevated CRP levels than those without endometriosis,^39^ it is not a reliable biomarker, and other inflammatory conditions more commonly elevate CRP levels. Indeed, a previous study^43^ reported that CRP levels were not associated with endometriosis risk. Accordingly, additional studies will be needed to investigate the association between inflammation and endometriosis and the potential association with psychiatric comorbidities.

Our genome-wide analysis identified *DGKB* rs12666606 as a pleiotropic variant between endometriosis and depression. Our enrichment analysis highlighted multiple pathways related to the function of its encoded protein, diacylglycerol kinase. This kinase converts diacylglycerol to phosphatidic acid and phosphatidate.^44^ Diacylglycerol kinase ζ enzymes are also closely involved in inflammatory and immune responses regulating nuclear factor–κB, which is a key component for inflammation, adaptive immune response, apoptosis, and oncogenesis.^45^ Furthermore, altered *DGKB* likely influences the breakdown of diacylglycerol and, hence, the production of estradiol,^46^ which is the primary hormone responsible for endometriosis progression.^47^ Accordingly, molecular pathways related to the *DGKB* gene could be informative of the pleiotropy associating endometriosis with other comorbid conditions, such as depression.

### Limitations

Our study has several limitations. First, the analysis was underpowered to test the association of endometriosis on psychiatric disorders and to investigate the association between eating disorders and endometriosis. Second, the phenotypic associations observed may be affected by the fact that individuals diagnosed with a chronic disease often have more hospital visits than those without any chronic disease. For instance, anxiety and depression could increase the diagnosis rate among people with endometriosis. However, because endometriosis is a highly undiagnosed disease, we believe that the large associations observed in the phenotypic analysis are unlikely to be driven by this potential bias. With respect to the UKB, a recent study^48^ demonstrated a sex-differential participation bias among UKB participants that was genetically correlated with educational attainment, risky behaviors, cannabis use, and body mass index. However, this bias was not genetically correlated with gynecological phenotypes, such as age at menarche and age at menopause; other female-specific disorders, such as breast cancer; and disorders with symptoms shared with endometriosis, such as irritable bowel syndrome.^48^ Although these data could support that endometriosis should not have a major association with sex-differential participation bias in the UKB, we cannot exclude that endometriosis prevalence may be misrepresented in the UKB. Third, we investigated only individuals of European descent because of the very limited sample size of other ancestry groups in UKB. Future studies will need to leverage more diverse samples to assess the transferability of the phenotypic and genetic associations across ancestry groups.

## Conclusions

In this genetic association study, eating disorders, depression, and anxiety were associated with endometriosis even after accounting for several comorbid conditions, including chronic pain. These phenotypic associations were in line with the genetic correlation of endometriosis with depression, anxiety, and eating disorders. In addition to possible shared associations, both depression and anxiety may be associated with the risk of endometriosis, although we lacked the statistical power to test this. To our knowledge, this is the first large-scale study to provide genetic and phenotypic evidence of the processes underlying the psychiatric comorbidities of endometriosis. This study contributes to the increasing evidence that endometriosis is a systemic disease that affects women’s mental and physical health.

## Notes

Supplement 1. **eMethods.** Supplemental Methods **eTable 1.** Sample Size Distribution of the Psychiatric Traits Investigated **eTable 2.** Summary of the Multiple Regression Model for the Phenotypic Association of Endometriosis With Depression, Anxiety, and Eating Disorders (ED) **eTable 3.** LD Independent Genome-Wide Significant Variants kin the Endometriosis GWAS Generated From UK Biobank Data **eTable 4.** Descriptive Statistics and SNV Heritability of the GWASs Generated From UK Biobank Data Using LDSC **eTable 5.** Genetic Correlation Among Endometriosis and Psychiatric Traits Using SCORE and LDSC and Genetic Correlation Between the Same Traits in Different Cohorts Using LDSC **eTable 6.** Details of the Polygenic Risk Score Analysis Across All P-Value Thresholds and Traits **eTable 7.** Results of the Bidirectional One-Sample Mendelian Randomization With Power Analysis and Diagnostic Tests **eTable 8.** Results With a Theta P-Value of <0.05 of the SNV-Level Pleiotropy Analysis **eTable 9.** Gene-Based Analysis for R < 0.05 SNVs in the Pleiotropy Analysis Using the Versatile Gene-Based Association Study (VEGAS) 2 Tool **eTable 10.** Pathway Analysis Based on the Gene-Based Analysis Using the Versatile Gene-Based Association Study (VEGAS) 2 Tool **eTable 11.** Association of the rs1266606 Variant With Sex-Stratified Phenotypic Traits in the UK Biobank **eTable 12.** Association of the rs1266606 Variant With Phenotypic Traits Available From the GWAS Atlas

Supplement 2.
Data Sharing Statement
