# Supplementary material for: Epidemiologic and Genetic Associations of Endometriosis With Depression, Anxiety, and Eating Disorders
Source: JAMA Netw Open. 2023 Jan 18;6(1):e2251214. doi: 10.1001/jamanetworkopen.2022.51214 (PMC9856929; doi:10.1001/jamanetworkopen.2022.51214)
Supplement: Supplement 1. — eMethods. Supplemental Methods eTable 1. Sample Size Distribution of the Psychiatric Traits Investigated eTable 2. Summary of the Multiple Regression Model for the Phenotypic Association of Endometriosis With Depression, Anxiety, and Eating Disorders (ED) eTable 3. LD Independent Genome-Wide Significant Variants kin the Endometriosis GWAS Generated From UK Biobank Data eTable 4. Descriptive Statistics and SNV Heritability of the GWASs Generated From UK Biobank Data Using LDSC eTable 5. Genetic Correlation Among Endometriosis and Psychiatric Traits Using SCORE and LDSC and Genetic Correlation Between the Same Traits in Different Cohorts Using LDSC eTable 6. Details of the Polygenic Risk Score Analysis Across All P-Value Thresholds and Traits eTable 7. Results of the Bidirectional One-Sample Mendelian Randomization With Power Analysis and Diagnostic Tests eTable 8. Results With a Theta P-Value of <0.05 of the SNV-Level Pleiotropy Analysis eTable 9. Gene-Based Analysis for R < 0.05 SNVs in the Pleiotropy Analysis Using the Versatile Gene-Based Association Study (VEGAS) 2 Tool eTable 10. Pathway Analysis Based on the Gene-Based Analysis Using the Versatile Gene-Based Association Study (VEGAS) 2 Tool eTable 11. Association of the rs1266606 Variant With Sex-Stratified Phenotypic Traits in the UK Biobank eTable 12. Association of the rs1266606 Variant With Phenotypic Traits Available From the GWAS Atlas [file jamanetwopen-e2251214-s001.pdf]

Supplementary Online Content

Koller D, Pathak GA, Wendt FR, et al. Epidemiologic and genetic associations of endometriosis with depression, anxiety, and eating disorders. *JAMA Netw Open*. 2023;6(1):e2251214. doi:10.1001/jamanetworkopen.2022.51214

eMethods. Supplemental Methods
eTable 1. Sample Size Distribution of the Psychiatric Traits Investigated
eTable 2. Summary of the Multiple Regression Model for the Phenotypic Association of Endometriosis With Depression, Anxiety, and Eating Disorders (ED)
eTable 3. LD Independent Genome-Wide Significant Variants kin the Endometriosis GWAS Generated From UK Biobank Data
eTable 4. Descriptive Statistics and SNV Heritability of the GWASs Generated From UK Biobank Data Using LDSC
eTable 5. Genetic Correlation Among Endometriosis and Psychiatric Traits Using SCORE and LDSC and Genetic Correlation Between the Same Traits in Different Cohorts Using LDSC
eTable 6. Details of the Polygenic Risk Score Analysis Across All P-Value Thresholds and Traits
eTable 7. Results of the Bidirectional One-Sample Mendelian Randomization With Power Analysis and Diagnostic Tests
eTable 8. Results With a Theta P-Value of <0.05 of the SNV-Level Pleiotropy Analysis
eTable 9. Gene-Based Analysis for R < 0.05 SNVs in the Pleiotropy Analysis Using the Versatile Gene-Based Association Study (VEGAS) 2 Tool
eTable 10. Pathway Analysis Based on the Gene-Based Analysis Using the Versatile Gene-Based Association Study (VEGAS) 2 Tool
eTable 11. Association of the rs1266606 Variant With Sex-Stratified Phenotypic Traits in the UK Biobank
eTable 12. Association of the rs1266606 Variant With Phenotypic Traits Available From the GWAS Atlas

This supplementary material has been provided by the authors to give readers additional information about their work.

eMethods. Supplemental Methods

Self-reported and ICD code phenotype definitions

The ICD10-N80 endometriosis diagnostic criteria include all endometriosis localizations including the ovaries, uterus, fallopian tube, pelvic peritoneum, pelvic sidewall, cul-de-sac, pelvic brim, intestine, rectovaginal septum and vagina, and ureter. The self-reported definition was derived from an assessment conducted by a trained nurse who explained the condition to the participants and registered their response. Before quality control, the total number of endometriosis cases in UKB was 5,308 for ICD codes, and 4,590 for self-reported criteria. Considering the overlap between ICD codes and self-reported diagnoses, in total 8,710 individuals were considered. After quality control, 5% of the sample was removed due to the exclusion of related individuals, and 8,276 cases were included in the subsequent analyses.

A total of 14,397 female depression cases were detected based on F32 ICD code (N=8,368), and self-reported depression (N=12,912). For anxiety, the 5,989 cases derived from the ICD10-F41 code (N=4,214), the ICD10-phecode 300 (F06, F34, F40, F41, F42, F43, F48, F93, F99, R45, N=4,901) and self-reported anxiety/panic attacks (N=3,832). We defined 591 female cases of eating disorders based on the ICD-10-R63 code for anorexia (N=504) and self-reported anorexia/bulimia/other eating disorder (N=284).

© 2023 Koller D et al. *JAMA Network Open.*

Logistic regression – phenotype association

Model 1 included age as covariate; Model 2 additionally adjusted for body mass index (BMI), Townsend deprivation index at recruitment, age at menarche, and length of menstrual cycle; Model 3 included all covariates in Model 2 and ever taken oral contraceptive pill, back pain for 3+ months, stomach/abdominal pain for 3+ months, medication for pain relief, constipation, heartburn, pain type(s) experienced in last month, ever diagnosed with irritable bowel syndrome, frequency of discomfort/pain in abdomen in last 3 months, discomfort/pain occurring only during menstrual bleed, abdominal discomfort/pain for 6 months or longer, frequency of discomfort/pain getting better or stopping after a bowel movement, more frequent bowel movements when abdominal discomfort/pain started, severity of current abdominal pain, and number of days (out of 10) with abdominal pain; Model 4 included all covariates in Model 3 together with the other psychiatric traits to investigate whether the association was due to the comorbidity among the three psychiatric disorders investigated (e.g., when endometriosis was tested with respect to anxiety, the Model 4 included depression and eating disorder as covariates together with the other covariates included in Model 3).

Data sources for genetic correlation and polygenic risk scoring

We used GWAS data from previous large-scale studies to investigate further the genetic effects linking endometriosis to depression, anxiety and eating disorders. To avoid sample overlap bias, the GWAS were selected only if they did not include the UKB cohort. For endometriosis, we selected genome-wide data generated by the FinnGen Study (Freeze 6)[1] considering an ICD-based definition (N_{cases} = 10,029, $N_{controls}$ = 81,593). For eating disorders, we used data generated from the Psychiatric Genomic Consortium (PGC), which meta-analyzed cohorts with a lifetime

© 2023 Koller D et al. *JAMA Network Open.*

diagnosis of anorexia nervosa, bulimia nervosa, or other eating disorders (3,495 cases and 10,982 controls)[2]. Although a larger eating-disorder GWAS is available[3], we could not use this dataset in the present study due to sample overlap with the UKB. For depression and anxiety, we used GWAS data generated from the Million Veteran Program (MVP)[4]. Briefly, anxiety was assessed using the GAD-2 scale in a total of 241,541 MVP participants[5], while MVP depression (N_{cases} = 83,810, $N_{controls}$ = 166,405) was defined by combining self-reported information, ICD codes, and a 2-item Patient Health Questionnaire-9 scale[4]. To investigate the potential role of inflammation in the psychiatric comorbidities of endometriosis, we also used GWAS data generated from the CHARGE (Cohorts for Heart and Aging Research in Genomic Epidemiology) consortium, which investigated circulating C-reactive protein (CRP) in 204,402 individuals[6].

One-sample Mendelian Randomization

Polygenic risk scoring: Prior to computing the PRS, SNVs were filtered to include those with imputation info score ≥ 0.8, and minor allele frequency ≥ 0.01. UKB individual-level data were used as target data for each phenotype. Analyses were adjusted for age and the first 10 within-ancestry PCs. Multiple genome-wide association p-value thresholds were considered (PT: 5×10^{-8}, 10^{-7}, 10^{-6}, 10^{-5}, 10^{-4}, .001, 0.05, 0.1, 0.2, 0.3, 0.4, 0.5, 1) for SNV inclusion and LD cut-off of R^2=0.1 within a 250-kb window as clumping criteria. The Major Histocompatibility Complex region of the genome was excluded because of its complex LD structure.

Sensitivity analyses: To verify that our analysis does not violate the MR assumptions, we conducted sensitivity analyses specifically designed for one-sample MR studies. Specifically, weak-instrument and Wu-Hausman tests were performed to evaluate whether the instruments

© 2023 Koller D et al. *JAMA Network Open.*

have a low correlation with the explanatory variable and whether the instrumental variables are as consistent as the ordinary least squares, respectively. A power analysis was also performed using the mRnd online tool[7]. The R^2 for each variant was calculated using the following formula: $R^2 = 2 \times Beta^2 \times MAF \times (1 - MAF)$, where MAF is the minor allele frequency in the UKB European ancestry participants.

Phenome-wide association study: To characterize further the rs12666606 pleiotropic variant, we performed a phenome-wide association study (PheWAS) of 3,287 phenotypes assessed in up to 194,174 female UKB participants (eTable 11 in the Supplement). Additionally, we also explored rs12666606 associations in the GWAS atlas[8]. We applied False Discovery Rate (FDR; q <0.05)[9] accounting for the number of phenotypes tested to identify associations surviving multiple testing correction.

eResults

Phenome-wide association study

Considering female UKB participants (N=194,174), 160 traits were nominally associated with *DGKB**rs12666606. Although no association survived multiple testing correction (FDR q<0.05, eTable 11 in the Supplement), the top results included enterocolitis due to *Clostridium difficile* (beta=0.001, p=4×10^{-4}), dorsalgia (beta=0.006, p=5×10^{-4}), maximum heart rate during fitness test (beta=0.084, p=8×10^{-4}), other and unspecified granulomatous disorders of skin and subcutaneous tissue (beta=0.001, p=8×10^{-4}), zumenon 1mg tablet (beta=0.001, p=9×10^{-4}), and low back pain

© 2023 Koller D et al. *JAMA Network Open.*

(beta=0.004, p=0.001). Considering sex-combined data available from the GWAS atlas[8], we did not identify any association surviving multiple testing correction (eTable 12 in Supplement 2).

eReferences

1. Mitja I. Kurki JK, Priit Palta, et al. FinnGen: Unique genetic insights from combining isolated population and national health register data. *medRxiv*. 2022;doi:10.1101/2022.03.03.22271360

2. Duncan L, Yilmaz Z, Gaspar H, et al. Significant Locus and Metabolic Genetic Correlations Revealed in Genome-Wide Association Study of Anorexia Nervosa. *Am J Psychiatry*. Sep 1 2017;174(9):850-858. doi:10.1176/appi.ajp.2017.16121402

3. Watson HJ, Yilmaz Z, Thornton LM, et al. Genome-wide association study identifies eight risk loci and implicates metabo-psychiatric origins for anorexia nervosa. *Nat Genet*. Aug 2019;51(8):1207-1214. doi:10.1038/s41588-019-0439-2

4. Levey DF, Stein MB, Wendt FR, et al. Bi-ancestral depression GWAS in the Million Veteran Program and meta-analysis in >1.2 million individuals highlight new therapeutic directions. *Nat Neurosci*. Jul 2021;24(7):954-963. doi:10.1038/s41593-021-00860-2

5. Levey DF, Gelernter J, Polimanti R, et al. Reproducible Genetic Risk Loci for Anxiety: Results From approximately 200,000 Participants in the Million Veteran Program. *Am J Psychiatry*. Mar 1 2020;177(3):223-232. doi:10.1176/appi.ajp.2019.19030256

6. Ligthart S, Vaez A, Vosa U, et al. Genome Analyses of >200,000 Individuals Identify 58 Loci for Chronic Inflammation and Highlight Pathways that Link Inflammation and Complex Disorders. *Am J Hum Genet*. Nov 1 2018;103(5):691-706. doi:10.1016/j.ajhg.2018.09.009

7. Brion MJ, Shakhbazov K, Visscher PM. Calculating statistical power in Mendelian randomization studies. *Int J Epidemiol*. Oct 2013;42(5):1497-501. doi:10.1093/ije/dyt179

8. Watanabe K, Stringer S, Frei O, et al. A global overview of pleiotropy and genetic architecture in complex traits. *Nat Genet*. Sep 2019;51(9):1339-1348. doi:10.1038/s41588-019-0481-0

9. Benjamini YH, Y. Controlling the False Discovery Rate: A Practical and Powerful Approach to Multiple Testing. *Journal of the Royal Statistical Society: Series B (Methodological)*. 1995;doi:10.1111/j.2517-6161.1995.tb02031.x

© 2023 Koller D et al. *JAMA Network Open.*

eTable 1. Sample Size Distribution of the Psychiatric Traits Investigated

Trait	Detailed description	UKB phenotype code	ICD codes included	Ncases among endometriosis cases	Ncases
Anorexia	Anorexia, loss of appetite. Exludes: anorexia nervosa (F50.0), loss of appetite of nonorganic origin (F50.89), bulimia not otherwise specified (F50.2).	260.6	R63	23	504
Anxiety disorders	F06: Anxiety disorder due to known physiological condition, F34: persistent mood disorders, F40: phobix anxiety disorders, F41: other anxiety disorders, F42: obsessive-compulsive disorder, F43: reaction to severe stress, and adjustment disorders, F48: Other nonpsychotic mental	300	F06, F34, F40, F41, F42, F43, F48, F93, F99, R45	165	4901

© 2023 Koller D et al. *JAMA Network Open.*

	disorders, F93: Separation anxiety disorder of childhood, F99: Mental disorder, not otherwise specified, R45: Symptoms and signs involving emotional state.				
Diagnoses - main ICD10: F41 Other anxiety disorders	Other anxiety disorders: Panic disorder, Generalized anxiety disorder, Other mixed anxiety disorders, Other specified anxiety disorders, anxiety disorder unspecified.	F41	F41	245	4214
Diagnoses - main ICD10: F32 Depressive episode	Depressive episode: Major depressive disorder, single episode, mild, Major depressive disorder, single episode, moderate, Major depressive disorder, single episode, severe without psychotic features, Major depressive disorder, single	F32	F32	434	8368

© 2023 Koller D et al. *JAMA Network Open.*

	episode, severe with psychotic features, Major depressive disorder, single episode, in partial remission, Major depressive disorder, single episode, in full remission, Other depressive episodes: Premenstrual dysphoric disorder, Other specified depressive episodes, Major depressive disorder, single episode, unspecified, Depression, unspecified.				
Non-cancer illness code, self-reported: anorexia/bulimia/other eating disorder	Code for non-cancer illness. If the participant was uncertain of the type of illness they had had, then they described it to the interviewer (a trained nurse) who attempted to place it within the coding tree. If the illness	20002_1470	NA	10	284

© 2023 Koller D et al. *JAMA Network Open.*

	could not be located in the coding tree then the interviewer entered a free-text description of it. These free-text descriptions were subsequently examined by a doctor and, where possible, matched to entries in the coding tree. Free-text descriptions which could not be matched with very high probability have been marked as "unclassifiable".				
Non-cancer illness code, self-reported: anxiety/panic attacks	Code for non-cancer illness. If the participant was uncertain of the type of illness they had had, then they described it to the interviewer (a trained nurse) who attempted to place it within the coding tree. If the illness could not be located	20002_1287	NA	85	3832

© 2023 Koller D et al. *JAMA Network Open.*

	in the coding tree then the interviewer entered a free-text description of it. These free-text descriptions were subsequently examined by a doctor and, where possible, matched to entries in the coding tree. Free-text descriptions which could not be matched with very high probability have been marked as "unclassifiable".				
Non-cancer illness code, self-reported: depression	Code for non-cancer illness. If the participant was uncertain of the type of illness they had had, then they described it to the interviewer (a trained nurse) who attempted to place it within the coding tree. If the illness could not be located in the coding tree	20002_1286	NA	419	12912

© 2023 Koller D et al. *JAMA Network Open.*

	then the interviewer entered a free-text description of it. These free-text descriptions were subsequently examined by a doctor and, where possible, matched to entries in the coding tree. Free-text descriptions which could not be matched with very high probability have been marked as "unclassifiable".				
**Combined trait**	**Ncases**	**Overlap between ICD and self-reported diagnoses**			
Depression	14397	67%			
Anxiety	5989	46%			
Eating disorders	591	75%			

© 2023 Koller D et al. *JAMA Network Open.*

eTable 2. Summary of the Multiple Regression Model for the Phenotypic Association of Endometriosis With Depression, Anxiety, and Eating Disorders (ED)
The estimates are compared between models.

**Model 1**	**Estimate**	**SE**	**z value**	**P**	**OR**	**2.5%**	**97.5%**
Depression	1.486	0.040	36.692	<2e-16	4.536	4.419	4.784
Anxiety	1.209	0.063	19.048	<2e-16	3.350	2.958	3.794
ED	1.614	0.197	8.203	<2e-16	5.021	3.415	7.384
**Model 2**	**Estimate**	**SE**	**z value**	**P**	**OR**	**2.5%**	**97.5%**
Depression	1.421	0.041	34.617	<2e-16	4.141	3.821	4.488
Anxiety	1.150	0.064	17.979	<2e-16	3.157	2.785	3.578
ED	1.590	0.198	8.014	1.11E-15	4.906	3.325	7.238
**Model 3**	**Estimate**	**SE**	**z value**	**P**	**OR**	**2.5%**	**97.5%**
Depression	1.296	0.042	31.055	<2e-16	3.653	3.367	3.965
Anxiety	0.993	0.065	15.307	<2e-16	2.700	2.378	3.067
ED	1.399	0.201	6.948	3.71E-12	4.053	2.731	6.015
**Model 4**	**Estimate**	**SE**	**z value**	**P**	**OR**	**2.5%**	**97.5%**
Depression	1.319	0.050	26.181	<2e-16	3.609	3.322	3.921
Anxiety	0.960	0.066	14.609	<2e-16	2.612	2.296	2.971
ED	1.079	0.207	5.220	1.79E-07	2.943	1.962	4.414

Model 1 covariates: age.
Model 2 covariates: age, BMI, socioeconomic status, age at menarche, length of menstrual cycle.
Model 3 covariates: model 2 covariates + ever taken oral contraceptive pill, medication for pain relief, constipation, heartburn, irritable bowel syndrome, pain phenotypes.
Model 4 covariates: model 3 covariates + other psychiatric diagnoses.

© 2023 Koller D et al. *JAMA Network Open.*

eTable 3. LD Independent Genome-Wide Significant Variants kin the Endometriosis GWAS Generated From UK Biobank Data

**SNV**	**Chromosome**	**Position**	**Allele 1**	**Allele 2**	**OR**	**P value**
rs7521902	1	22490724	A	C	1.125	2.50E-09
rs6432220	2	11710716	A	G	0.901	3.57E-08
rs10013228	4	55997340	G	A	0.898	3.39E-08
rs12173791	6	151824449	A	G	1.182	1.54E-08
rs12110523	6	152556258	C	T	1.132	3.58E-09
rs4071558	11	30344591	T	C	0.842	2.35E-12

© 2023 Koller D et al. *JAMA Network Open.*

eTable 4. Descriptive Statistics and SNV Heritability of the GWASs Generated From UK Biobank Data Using LDSC

**Trait**	**h2**	**se**	**z score**	**lambda GC**	**Mean χ2**	**Intercept**	**se**	**Ratio**	**se**
Endometriosis	0.0859	0.015	5.72666667	1.0618	1.0769	1.0192	-0.0071	0.2498	-0.0923
Depression	0.0295	0.0025	11.8000	1.1270	1.1367	1.0035	0.0073	0.0260	0.0533
Anxiety	0.0124	0.0022	5.6364	1.0649	1.0658	1.0069	0.0067	0.1050	0.1014
ED	0.0001	0.0018	0.0556	1.0135	1.0097	1.0091	0.0060	0.9343	0.6154

© 2023 Koller D et al. *JAMA Network Open.*

eTable 5. Genetic Correlation Among Endometriosis and Psychiatric Traits Using SCORE and LDSC and Genetic Correlation Between the Same Traits in Different Cohorts Using LDSC

**SCORE - without psychiatric covariates**					
**p1**	**p2**	**rg**	**se**	**z**	**p value**
UKB endometriosis	UKB depression	0.364	0.061	5.934	1.5E-09
UKB endometriosis	UKB anxiety	0.334	0.079	4.229	1.17E-05
UKB endometriosis	UKB eating disorders	0.609	0.310	1.962	0.025
**SCORE - with covariates**					
**p1**	**p2**	**rg**	**se**	**z**	**p value**
UKB endometriosis	UKB depression	0.341	0.068	4.989	3.04E-07
UKB endometriosis	UKB anxiety	0.261	0.099	2.644	4.10E-03
UKB endometriosis	UKB eating disorders	0.589	0.381	1.546	0.061
**LDSC**					
**p1**	**p2**	**rg**	**se**	**z**	**p value**
UKB endometriosis	UKB depression	0.362	0.101	3.581	3.00E-04
UKB endometriosis	UKB anxiety	0.375	0.129	2.895	3.80E-03
**LDSC - between different cohorts**					
**p1**	**p2**	**rg**	**se**	**z**	**p value**
UKB endometriosis	FinnGen endometriosis	1.169	0.143	8.188	2.66E-16
UKB depression	MVP depression	0.814	0.030	26.919	1.32E-159
UKB anxiety	MVP anxiety	0.724	0.106	6.825	8.78E-12
UKB CRP	CHARGE CRP	0.990	0.029	34.515	4.77E-261

© 2023 Koller D et al. *JAMA Network Open.*

eTable 6. Details of the Polygenic Risk Score Analysis Across All P-Value Thresholds and Traits

Phenotype	P-value Threshold	R^2	P-value	Coefficient	Standard Error	Number of SNVs
Endometriosis	0.0001	7.71E-05	0.057	1.61	0.845	2
	0.001	2.12E-05	0.318	2.59	2.591	19
	0.05	1.13E-04	0.021	38.23	16.570	438
	0.1	4.23E-05	0.158	31.72	22.491	730
	0.2	5.34E-05	0.113	49.29	31.125	1170
	0.3	2.57E-05	0.272	42.38	38.560	1548
	0.4	1.91E-05	0.344	42.50	44.882	1862
	0.5	2.59E-05	0.270	55.72	50.539	2133
	1	3.48E-05	0.201	87.87	68.746	2953
Depression	5E-08	2.86E-03	1.58E-59	139.97	8.602	165
	1E-07	2.91E-03	1.45E-60	150.99	9.197	187
	1E-06	4.81E-03	2.76E-99	299.92	14.181	354
	1E-05	8.01E-03	9.93E-164	638.04	23.398	726
	0.0001	0.0125	1.94E-253	1385.02	40.731	1656
	0.001	0.0233	<1E-300	3788.96	81.604	4718
	0.05	0.0596	<1E-300	25093.00	342.150	46124
	0.1	0.0672	<1E-300	35427.00	456.564	71675
	0.2	0.0735	<1E-300	50212.60	619.959	110765
	0.3	0.0761	<1E-300	61774.40	750.719	142026
	0.4	0.0776	<1E-300	71602.90	862.293	167861
	0.5	0.0783	<1E-300	80020.30	959.612	189776
	1	0.0790	<1E-300	106541.00	1272.390	255350
Anxiety	5E-08	3.99E-05	0.132	3.33	2.211	5
	1E-07	2.21E-05	0.262	2.75	2.453	6
	1E-06	1.38E-05	0.376	-3.33	3.759	18

© 2023 Koller D et al. *JAMA Network Open.*

	1E-05	1.16E-05	0.416	7.53	9.254	82
	0.0001	2.22E-04	3.79E-04	82.78	23.289	398
	0.001	8.21E-04	8.20E-12	415.13	60.735	2172
	0.05	1.37E-03	1.09E-18	3012.27	341.327	44499
	0.1	1.18E-03	2.60E-16	3838.62	468.673	74953
	0.2	1.16E-03	4.72E-16	5333.57	656.968	124148
	0.3	1.13E-03	1.07E-15	6494.30	809.955	165139
	0.4	1.21E-03	1.19E-16	7808.00	942.474	199929
	0.5	1.18E-03	2.35E-16	8699.42	1060.580	229796
	1	1.17E-03	3.69E-16	11853.30	1454.690	321526
**Anorexia nervosa**	5E-08	8.26E-05	0.367	0.53	0.593	1
	1E-07	1.06E-04	0.307	0.90	0.882	2
	1E-06	6.94E-06	0.794	-0.34	1.312	4
	1E-05	2.15E-04	0.145	5.55	3.811	27
	0.0001	2.39E-04	0.125	16.72	10.896	182
	0.001	5.51E-05	0.462	24.78	33.661	1362
	0.05	1.06E-03	1.25E-03	656.72	203.521	33521
	0.1	8.63E-04	3.57E-03	818.01	280.710	56954
	0.2	8.73E-04	3.37E-03	1154.87	393.973	94980
	0.3	9.23E-04	2.59E-03	1465.57	486.485	126719
	0.4	9.13E-04	2.73E-03	1695.11	565.641	153333
	0.5	9.22E-04	2.62E-03	1874.58	623.079	175695
	1	1.14E-03	8.36E-04	2811.07	841.479	242016
**C-reactive protein**	5E-08	0.0155	<1E-300	278.01	5.019	170
	1E-07	0.0158	<1E-300	304.56	5.445	187
	1E-06	0.0162	<1E-300	385.99	6.806	248
	1E-05	0.0170	<1E-300	588.10	10.121	398
	0.0001	0.0166	<1E-300	1021.27	17.768	801

© 2023 Koller D et al. *JAMA Network Open.*

	0.001	0.0152	<1E-300	2572.51	46.887	2638
	0.05	0.0104	<1E-300	14539.10	321.665	40722
	0.1	9.75E-03	<1E-300	19760.20	450.740	67338
	0.2	8.97E-03	<1E-300	26773.10	636.647	109405
	0.3	8.64E-03	<1E-300	32363.50	784.558	143480
	0.4	8.49E-03	<1E-300	37131.30	908.259	171631
	0.5	8.41E-03	<1E-300	41465.70	1018.850	195825
	1	8.36E-03	<1E-300	55369.90	1364.930	266393

© 2023 Koller D et al. *JAMA Network Open.*

eTable 7. Results of the Bidirectional One-Sample Mendelian Randomization With Power Analysis and Diagnostic Tests

Exposure -> Outcome	Estimate	SE	t-value	P-value	OR	2.5%CI	97.5%CI	R2	Power	NCP	F-statistic
**Endometriosis -> Depression**	0.511	0.782	0.653	0.514	1.667	0.360	2.974	1.46E-04	0.11	0.52	14.38
**Endometriosis -> Anxiety**	0.423	0.532	0.796	0.426	1.527	0.539	2.516	1.46E-04	0.09	0.33	14.38
**Endometriosis -> ED**	0.043	0.159	0.27	0.787	1.044	0.764	1.324	1.46E-04	0.05	0	14.38
**Anxiety -> Endometriosis**	0.330	0.104	3.171	1.52E-03	1.391	1.134	1.647	2.40E-03	1	70.84	582.09
**Depression -> Endometriosis**	0.087	0.007	13.027	<2e-16	1.091	1.077	1.106	0.015	1	28.33	1735
**ED -> Endometriosis**	-1.464	0.929	-1.575	0.115	0.733	0.037	1.428	1.06E-04	0.05	0.02	2.53
**CRP -> Endometriosis**	-4.13E-04	7.00E-04	-0.590	0.555	1.000	0.998	1.001	0.043	1	59.95	492.74

Diagnostic tests		df1	df2	F statistic	p-value
**Endometriosis -> Anxiety**	**Weak instruments**	1	194527	5.328	0.021
	**Wu-Hausman**	1	194526	0.645	0.422
**Endometriosis -> Depression**	**Weak instruments**	1	194527	5.328	0.021
	**Wu-Hausman**	1	194526	0.375	0.54
**Endometriosis -> ED**	**Weak instruments**	1	194527	5.328	0.021
	**Wu-Hausman**	1	194526	0.074	0.785
**Anxiety -> Endometriosis**	**Weak instruments**	1	194527	7695	<2e-16
	**Wu-Hausman**	1	194526	120.5	<2e-16
**Depression -> Endometriosis**	**Weak instruments**	1	194527	78.01	<2e-16
	**Wu-Hausman**	1	194526	10.39	1.27E-03

© 2023 Koller D et al. *JAMA Network Open.*

**ED -> Endometriosis**	**Weak instruments**	1	194527	11.153	8.39E-04
	**Wu-Hausman**	1	194526	3.188	0.074
**CRP -> Endometriosis**	**Weak instruments**	1	194527	3376.287	<2e-16
	**Wu-Hausman**	1	194526	2.462	0.117

© 2023 Koller D et al. *JAMA Network Open.*

eTable 8. Results With a Theta P-Value of <0.05 of the SNV-Level Pleiotropy Analysis
Nominally Significant (Theta P-Value <0.05) Variants Between Endometriosis And Psychiatric Traits Are Highlighted In Green.

SNV	Endometriosis z-score	Depression z-score	Anxiety z-score	Eating disorders z-score	r	angle	r p-value	r q-value	theta p-value	theta q-value
rs12666606	-9.455	8.096	0.006	0.102	12.448	2.124	2.26E-34	9.25E-32	5.56E-08	4.95E-04
rs28699022	0.006	15.451	0.018	-6.574	16.792	1.207	5.92E-62	3.24E-59	1.31E-06	5.83E-03
rs12294968	-0.008	-0.058	81.174	-27.434	85.685	0.978	0	0	2.47E-06	7.34E-03
rs51500	-0.032	-11.144	-0.013	-6.510	12.906	1.586	6.74E-37	2.86E-34	3.31E-06	7.37E-03
rs2864004	-0.038	6.037	0.010	-11.123	12.656	1.492	1.66E-35	6.91E-33	2.04E-05	0.036
rs2159023	-6.260	-0.072	0.210	-6.397	8.953	2.325	3.94E-18	1.17E-15	4.90E-05	0.073
rs2286286	0.012	0.028	-12.069	-5.266	13.167	1.234	2.24E-38	9.71E-36	1.53E-04	0.194

© 2023 Koller D et al. *JAMA Network Open.*

eTable 9. Gene-Based Analysis for R < 0.05 SNVs in the Pleiotropy Analysis Using the Versatile Gene-Based Association Study (VEGAS) 2 Tool

The rs12666606 shared SNV between endometriosis and depression was mapped to the DGKB gene. Genes with a p-value < 0.05 are listed in the table.

Chromosome	Gene	N SNVs	Start	Stop	Test statistic	p value	q value	Top SNV	Top SNV p value
7	DGKB	6	14184673	14881075	29.511	1.00E-06	8.25E-04	rs12666606	5.56E-08
11	DLG2	7	83166055	85338314	22.190	1.60E-05	6.60E-03	rs12294968	2.47E-06
4	CRMP1	2	5822490	5894810	14.335	2.95E-04	0.081	rs2286286	1.53E-04
12	BTBD11	2	107712196	108053419	8.207	0.011	1	rs4964583	4.17E-03
13	HS6ST3	3	96743092	97491816	7.501	0.017	1	rs1927796	6.17E-03
2	MREG	3	216807313	216878346	6.544	0.030	1	rs3770551	0.011
12	CNTN1	2	41086243	41466213	5.523	0.032	1	rs11178843	0.019

© 2023 Koller D et al. *JAMA Network Open.*

eTable 10. Pathway Analysis Based on the Gene-Based Analysis Using the Versatile Gene-Based Association Study (VEGAS) 2 Tool

Nominally significant pathways are shown.

Gene Ontology ID	N genes	p value	q value	Genes
GO:0004143 diacylglycerol kinase activity	2	6.37E-06	0.018	DGKG,DGKB
PANTHER MOLECULAR FUNCTION Kinase	3	2.59E-05	0.024	DGKG,DGKB,TPK1
GO:0007205 activation of protein kinase C activity by G-protein coupled receptor protein signaling pathway	3	2.59E-05	0.024	GAP43,DGKG,DGKB
REACTOME EFFECTS OF PIP2 HYDROLYSIS	5	2.24E-04	0.155	PRKCE,MGLL,DGKG,DGKB,PRKCH
GO:0019205 nucleobase nucleoside nucleotide kinase activity	3	3.28E-04	0.182	SPEF2,ADK,DLG2
GO:0032147 activation of protein kinase activity	6	5.38E-04	0.249	GAP43,MAP3K13,DGKG,DGKB,EGFR,INSR
REACTOME CRMPS IN SEMA3A SIGNALING	3	2.38E-03	0.943	PLXNA2,CRMP1,DPYSL2
REACTOME G ALPHA Q SIGNALLING EVENTS	9	4.42E-03	1	GNG4,PRKCE,TACR1,KALRN,MGLL,DGKG,DGKB,PRKCH,PLCB4
Panther Axon guidance mediated by semaphorins	4	6.05E-03	1	PLXNA2,ARHGEF3,CRMP1,DPYSL2
PANTHER BIOLOGICAL PROCESS Lipid metabolism	10	7.79E-03	1	PIK3CD,PLD5,DGKG,DGKB,SGMS1,PIK3C2G,ST8SIA1,PLCG2,PLCB4,SPTLC3
GO:0045860 positive regulation of protein kinase activity	11	0.013	1	DAB1,GAP43,MAP3K13,DGKG,MAP3K5,DGKB,EGFR,RELN,FGD4,MAP3K9,INSR
PANTHER BIOLOGICAL PROCESS Nucleoside, nucleotide and nucleic acid metabolism	5	0.013	1	ATP6V1G3,CRMP1,DDX4,DPYSL2,ADK
GO:0005819 spindle	5	0.019	1	RCC2,CRMP1,NEDD9,MAD1L1,MAP2K5
GO:0051347 positive regulation of transferase activity	12	0.021	1	DAB1,GAP43,MAP3K13,DGKG,MAP3K5,DGKB,EGFR,RELN,VAV2,FGD4,MAP3K9,INSR
GO:0045859 regulation of protein kinase activity	12	0.021	1	DAB1,GAP43,MAP3K13,DGKG,MAP3K5,DGKB,EGFR,RELN,FGD4,MAP3K9,PRKCA,INSR
GO:0033674 positive regulation of kinase activity	12	0.021	1	DAB1,GAP43,MAP3K13,DGKG,MAP3K5,DGKB,EGFR,RELN,VAV2,FGD4,MAP3K9,INSR

© 2023 Koller D et al. *JAMA Network Open.*

REACTOME GASTRIN CREB SIGNALLING PATHWAY VIA PKC AND MAPK	12	0.021	1	RPS6KA1,GNG4,PRKCE,TACR1,KALRN,MGLL,DGKG,DGKB,EGFR,PRKCH,PRKCA,PLCB4
REACTOME PLATELET ACTIVATION SIGNALING AND AGGREGATION	14	0.022	1	PLA2G4A,GNG4,AKT3,PRKCE,RAPGEF4,FN1,MGLL,DGKG,F13A1,DGKB,ABCC4,PRKCH,PLCG2,PRKCA
REACTOME SEMAPHORIN INTERACTIONS	6	0.025	1	PLXNA2,FARP2,CRMP1,SEMA5A,DPYSL2,SEMA6D
GO:0031225 anchored to membrane	13	0.028	1	NEGR1,CNTN6,CNTN4,LSAMP,EFNA5,GOLGA7,DLG2,CNTN5,OPCML,CNTN1,GPC6,MDGA2,CDH13
GO:0043549 regulation of kinase activity	13	0.032	1	DAB1,GAP43,MAP3K13,DGKG,MAP3K5,DGKB,EGFR,RELN,VAV2,FGD4,MAP3K9,PRKCA,INSR
GO:0051338 regulation of transferase activity	13	0.032	1	DAB1,GAP43,MAP3K13,DGKG,MAP3K5,DGKB,EGFR,RELN,VAV2,FGD4,MAP3K9,PRKCA,INSR

© 2023 Koller D et al. *JAMA Network Open.*

eTable 11. Association of the rs1266606 Variant With Sex-Stratified Phenotypic Traits in the UK Biobank
Nominally significant associations are listed.

Phenotype	Phenotype code	beta	se	t statistic	p value	q value
Enterocolitis due to Clostridium difficile	C DIFFICILE ENTEROCOLITIS	0.0008	0.0002	3.5669	0.0004	0.5144
Diagnoses - main ICD10: M54 Dorsalgia	M54	0.0055	0.0016	3.4813	0.0005	0.5144
Dorsalgia	M13 DORSALGIA	0.0055	0.0016	3.4813	0.0005	0.5144
Maximum heart rate during fitness test	6033 irnt	0.0844	0.0252	3.3507	0.0008	0.5144
Other and unspecified granulomatous disorders of skin and subcutaneous tissue	L12 GRANULOMASKINNAS	0.0008	0.0002	3.3437	0.0008	0.5144
Treatment/medication code: zumenon 1mg tablet	20003 1140928878	0.0008	0.0002	3.3082	0.0009	0.5144
Low back pain	M13 LOWBACKPAIN	0.0040	0.0013	3.2373	0.0012	0.5665
Fractured bone site(s): Leg	6151 2	0.0023	0.0008	3.0191	0.0025	0.9833
Destinations on discharge from hospital (recoded): Transfer within NHS provider: Surgical specialty	41248 6004	0.0013	0.0005	2.9402	0.0033	0.9833
Used an inhaler for chest within last hour	3090	-0.0026	0.0009	-2.9247	0.0034	0.9833
Forced expiratory volume in 1-second (FEV1), predicted	20153 irnt	-0.0285	0.0100	-2.8472	0.0044	0.9833
Radial styloid tenosynovitis [de Quervain]	M13 DEQUERVAIN	0.0009	0.0003	2.8369	0.0046	0.9833
Diagnoses - main ICD10: O72 Postpartum haemorrhage	O72	0.0011	0.0004	2.8357	0.0046	0.9833
Vocal cord dysfunction	VOCALCORDDYS	0.0008	0.0003	2.7906	0.0053	0.9833
Job SOC coding: Scientific researchers	22617 2321	0.0053	0.0019	2.7748	0.0055	0.9833
6mm weak meridian angle (left)	5102 irnt	0.0625	0.0230	2.7196	0.0065	0.9833
Workplace very dusty: Sometimes	22609 1	-0.0247	0.0091	-2.7079	0.0068	0.9833
Treatment/medication code: metoclopramide	20003 1140879494	0.0010	0.0004	2.6890	0.0072	0.9833

© 2023 Koller D et al. *JAMA Network Open.*

Bacterial intestinal infections, IBD co-morbidities	IBD BACT INTEST INFECTIONS	0.0013	0.0005	2.6850	0.0073	0.9833
6mm cylindrical power angle (left)	5113 irnt	0.0616	0.0230	2.6802	0.0074	0.9833
Treatment/medication code: qvar 50 inhaler	20003 1141167594	-0.0017	0.0007	-2.6641	0.0077	0.9833
Job coding: scientific researcher, scientific officer, medical research associate, experimental officer	22601 23213026	0.0051	0.0019	2.6621	0.0078	0.9833
Worried most days during period of worst anxiety	20538	0.0226	0.0087	2.6028	0.0093	0.9833
Illnesses of adopted siblings: None of the above (group 1)	20114 100	-0.1580	0.0610	-2.5888	0.0098	0.9833
Diagnoses - main ICD10: R91 Abnormal findings on diagnostic imaging of lung	R91	-0.0013	0.0005	-2.5790	0.0099	0.9833
Breast disease (not cancer)	20002 1364	-0.0008	0.0003	-2.5752	0.0100	0.9833
Job coding: chef, cook, caterer	22601 54342896	-0.0035	0.0013	-2.5745	0.0100	0.9833
Workplace very noisy: Often	22606 2	-0.0188	0.0073	-2.5698	0.0102	0.9833
Diagnoses - main ICD10: A04 Other bacterial intestinal infections	A04	0.0013	0.0005	2.5608	0.0104	0.9833
Vitamin and mineral supplements: Vitamin D	6155 4	0.0055	0.0021	2.5557	0.0106	0.9833
Diagnoses - main ICD10: H71 Cholesteatoma of middle ear	H71	0.0007	0.0003	2.5457	0.0109	0.9833
Viral hepatitis	AB1 VIRAL HEPATITIS	0.0006	0.0002	2.5447	0.0109	0.9833
Pork intake	1389	0.0174	0.0069	2.5435	0.0110	0.9833
Diagnoses - main ICD10: M17 Gonarthrosis [arthrosis of knee]	M17	-0.0043	0.0017	-2.5389	0.0111	0.9833
Gonarthrosis [arthrosis of knee](FG)	KNEE ARTHROSIS	-0.0043	0.0017	-2.5389	0.0111	0.9833
Ulcerative colitis, NAS	ULCERNAS	0.0017	0.0007	2.5242	0.0116	0.9833

© 2023 Koller D et al. *JAMA Network Open.*

Diagnoses - main ICD10: L92 Granulomatous disorders of skin and subcutaneous tissue	L92	0.0008	0.0003	2.5090	0.0121	0.9833
Granulomatous disorders of skin and subcutaneous tissue	L12 GRANULOMATOUSSKIN	0.0008	0.0003	2.5090	0.0121	0.9833
Average monthly red wine intake	4407	0.0702	0.0281	2.4985	0.0125	0.9833
Non-cancer illness code, self-reported: disc problem	20002 1532	0.0007	0.0003	2.4926	0.0127	0.9833
Breathing problems improved/stopped away from workplace or on holiday: No	22618 0	-0.0107	0.0043	-2.4916	0.0127	0.9833
Diagnoses - main ICD10: I47 Paroxysmal tachycardia	I47	0.0016	0.0007	2.4894	0.0128	0.9833
6mm regularity index (left)	5162 irnt	0.0681	0.0275	2.4795	0.0132	0.9833
Treatment/medication code: dihydrocodeine	20003 1140884464	-0.0015	0.0006	-2.4793	0.0132	0.9833
Spherical power (left)	5085 irnt	-0.0515	0.0210	-2.4572	0.0140	0.9916
Diagnoses - main ICD10: L73 Other follicular disorders	L73	-0.0007	0.0003	-2.4526	0.0142	0.9916
Other follicular disorders	L12 FOLICULAROTH	-0.0007	0.0003	-2.4526	0.0142	0.9916
Neutrophill percentage	30200 irnt	0.0239	0.0099	2.4142	0.0158	0.9916
Job coding: senior official in central government including members of the senior civil service and the equivalent in the diplomatic service, mps, meps	22601 11113200	0.0038	0.0016	2.4052	0.0162	0.9916
Spherical power (right)	5084 irnt	-0.0501	0.0209	-2.4008	0.0164	0.9916
Diagnoses - main ICD10: K05 Gingivitis and periodontal diseases	K05	0.0009	0.0004	2.3993	0.0164	0.9916
ECG, heart rate	5983 irnt	0.0611	0.0255	2.3978	0.0165	0.9916
Treatment/medication code: glucophage 500mg tablet	20003 1140874686	-0.0007	0.0003	-2.3897	0.0169	0.9916

© 2023 Koller D et al. *JAMA Network Open.*

Pulse wave Arterial Stiffness index	21021 irnt	-0.0410	0.0172	-2.3777	0.0174	0.9916
Job coding: librarian, assistant librarian	22601 24513314	0.0053	0.0022	2.3760	0.0175	0.9916
Other/unspecified dorsalgia	M13 DORSALGIANAS	0.0019	0.0008	2.3721	0.0177	0.9916
Eye problems/disorders: Injury or trauma resulting in loss of vision	6148 3	0.0046	0.0019	2.3677	0.0179	0.9916
Diagnoses - main ICD10: Z04 Examination and observation for other reasons	Z04	0.0009	0.0004	2.3500	0.0188	0.9916
Frequency of needing morning drink of alcohol after heavy drinking session in last year	20412	0.0072	0.0031	2.3494	0.0188	0.9916
Manic/hyper symptoms: I was more talkative than usual	6156 12	-0.0354	0.0152	-2.3382	0.0194	0.9916
Non-cancer illness code, self-reported: essential hypertension	20002 1072	-0.0015	0.0007	-2.3343	0.0196	0.9916
Bell's palsy	G6 BELLPA	-0.0007	0.0003	-2.3335	0.0196	0.9916
Target heart rate achieved	6034	0.0201	0.0086	2.3333	0.0196	0.9916
Job SOC coding: Senior officials in national government	22617 1111	0.0036	0.0016	2.3266	0.0200	0.9916
Job SOC coding: IT user support technicians	22617 3132	-0.0035	0.0015	-2.3259	0.0200	0.9916
Lymphocyte percentage	30180 irnt	-0.0229	0.0099	-2.3134	0.0207	0.9916
Type of meals eaten: Bought sandwiches	20089 463	-0.0163	0.0071	-2.3097	0.0209	0.9916
Job coding: it user support technician, help desk or helpline operator (computing), support technician (computing), systems support officer	22601 31323061	-0.0034	0.0015	-2.3070	0.0211	0.9916
Illnesses of siblings: Alzheimer's disease/dementia	20111 10	0.0019	0.0008	2.2987	0.0215	0.9916
Dried fruit intake	1319	-0.0183	0.0081	-2.2753	0.0229	0.9916
Treatment/medication code: epaderm ointment	20003 1140927730	-0.0005	0.0002	-2.2668	0.0234	0.9916

© 2023 Koller D et al. *JAMA Network Open.*

Night shifts worked: This type of shift pattern was not worked during job	22650 9	0.0177	0.0078	2.2665	0.0234	0.9916
Female-specific factors	2734	-0.0162	0.0072	-2.2643	0.0236	0.9916
Non-cancer illness code, self-reported: throat or larynx disorder	20002 1414	-0.0007	0.0003	-2.2573	0.0240	0.9916
Job SOC coding: Architects	22617 2431	-0.0026	0.0011	-2.2565	0.0240	0.9916
Sodium in urine	30530 irnt	0.0222	0.0099	2.2381	0.0252	0.9916
Endocrine, nutritional and metabolic diseases	IV ENDOCRIN NUTRIT	-0.0031	0.0014	-2.2355	0.0254	0.9916
Pulse wave peak to peak time	4196 irnt	0.0381	0.0171	2.2285	0.0259	0.9916
Ever had period of mania / excitability	20501	0.0071	0.0032	2.2276	0.0259	0.9916
Diagnoses - main ICD10: C53 Malignant neoplasm of cervix uteri	C53	-0.0007	0.0003	-2.2269	0.0260	0.9916
Delivery methods: Low forceps, not breech	41221 2	0.0189	0.0085	2.2260	0.0260	0.9916
Job SOC coding: Psychologists	22617 2212	-0.0029	0.0013	-2.2220	0.0263	0.9916
Age asthma diagnosed	3786 irnt	0.0613	0.0277	2.2156	0.0267	0.9916
Diagnoses - main ICD10: D70 Agranulocytosis	D70	0.0013	0.0006	2.2073	0.0273	0.9916
Illnesses of adopted siblings: High blood pressure	20114 8	0.1137	0.0516	2.2025	0.0279	0.9916
Leg fat-free mass (right)	23113 irnt	-0.0212	0.0097	-2.1849	0.0289	0.9916
Job coding: psychologist, psychometrist	22601 22123067	-0.0028	0.0013	-2.1815	0.0292	0.9916
Treatment/medication code: ramipril	20003 1140860806	0.0037	0.0017	2.1801	0.0293	0.9916
Leg predicted mass (right)	23114 irnt	-0.0211	0.0097	-2.1761	0.0296	0.9916
Mental health problems ever diagnosed by a professional: Anxiety, nerves or generalized anxiety disorder	20544 15	0.0138	0.0063	2.1754	0.0296	0.9916
Neutrophill count	30140 irnt	0.0214	0.0099	2.1571	0.0310	0.9916

© 2023 Koller D et al. *JAMA Network Open.*

Home area population density - urban or rural: Scotland - Accessible Small Town	20118 13	-0.0012	0.0006	-2.1552	0.0311	0.9916
Illnesses of mother: Chronic bronchitis/emphysema	20110 6	-0.0053	0.0025	-2.1544	0.0312	0.9916
Diagnoses - main ICD10: M89 Other disorders of bone	M89	-0.0011	0.0005	-2.1482	0.0317	0.9916
Diagnoses - main ICD10: K20 Oesophagitis	K20	0.0023	0.0011	2.1464	0.0318	0.9916
Frequency of heavy DIY in last 4 weeks	2624	0.0432	0.0202	2.1401	0.0324	0.9916
Fortified wine intake	100720	-0.0157	0.0073	-2.1374	0.0326	0.9916
Congenital malformations of heart and great arteries	CONGEN HEART ARTER	-0.0007	0.0003	-2.1368	0.0326	0.9916
Illnesses of adopted mother: None of the above (group 1)	20113 100	-0.1106	0.0517	-2.1380	0.0327	0.9916
Recent medication for COPD (Chronic Obstructive Pulmonary Disease)	22170	0.1858	0.0868	2.1405	0.0328	0.9916
Treatment/medication code: depo-provera 50mg/1ml injection	20003 1141166200	-0.0005	0.0002	-2.1327	0.0329	0.9916
Non-cancer illness code, self-reported: hypothyroidism/myxoedema	20002 1226	-0.0055	0.0026	-2.1235	0.0337	0.9916
Leg fat-free mass (left)	23117 irnt	-0.0206	0.0097	-2.1189	0.0341	0.9916
Illnesses of adopted siblings: Heart disease	20114 1	0.0820	0.0387	2.1171	0.0345	0.9916
Leg predicted mass (left)	23118 irnt	-0.0205	0.0097	-2.1051	0.0353	0.9916
Diagnoses - main ICD10: Z51 Other medical care	Z51	-0.0005	0.0003	-2.1030	0.0355	0.9916
Average monthly champagne plus white wine intake	4418	0.0627	0.0299	2.0997	0.0358	0.9916
Actions taken following self-harm: Receive help from friends / family / neighbours	20554 6	0.0044	0.0021	2.0963	0.0361	0.9916
Diagnoses - main ICD10: M21 Other acquired deformities of limbs	M21	-0.0009	0.0004	-2.0939	0.0363	0.9916

© 2023 Koller D et al. *JAMA Network Open.*

Ever self-harmed	20480	0.0080	0.0038	2.0938	0.0363	0.9916
Illnesses of siblings: Chronic bronchitis/emphysema	20111 6	0.0040	0.0019	2.0931	0.0363	0.9916
Diagnoses - main ICD10: K62 Other diseases of anus and rectum	K62	-0.0039	0.0019	-2.0928	0.0364	0.9916
COPD, early/later onset	COPD EARLYANDLATER	0.0014	0.0007	2.0913	0.0365	0.9916
Whole body fat-free mass	23101 irnt	-0.0203	0.0097	-2.0893	0.0367	0.9916
Diagnoses - main ICD10: R18 Ascites	R18	0.0007	0.0003	2.0884	0.0368	0.9916
Never eat eggs, dairy, wheat, sugar: Dairy products	6144 2	-0.0031	0.0015	-2.0875	0.0368	0.9916
Current employment status: In paid employment or self-employed	6142 1	-0.0082	0.0039	-2.0848	0.0371	0.9916
Number of triplets attempted (left)	4269	0.0089	0.0043	2.0828	0.0373	0.9916
Hidradenitis suppurativa	L12 HIDRADENITISSUP	-0.0005	0.0002	-2.0780	0.0377	0.9916
Treatment/medication code: airomir 100micrograms cfc-free inhaler	20003 1140917034	-0.0005	0.0002	-2.0777	0.0377	0.9916
Diagnoses - main ICD10: Z48 Other surgical follow-up care	Z48	0.0010	0.0005	2.0714	0.0383	0.9916
Nitrogen dioxide air pollution; 2007	24018 irnt	0.0203	0.0098	2.0709	0.0384	0.9916
Diagnoses - main ICD10: N12 Tubulo-interstitial nephritis, not specified as acute or chronic	N12	0.0010	0.0005	2.0684	0.0386	0.9916
Manic/hyper symptoms: I was more active than usual	6156 11	0.0335	0.0163	2.0625	0.0392	0.9916
Frequency of inability to stop worrying during worst period of anxiety	20539	0.0412	0.0200	2.0595	0.0395	0.9916
Manic/hyper symptoms: None of the above	6156 100	0.0384	0.0187	2.0557	0.0398	0.9916
Diagnoses - main ICD10: Q21 Congenital malformations of cardiac septa	Q21	-0.0006	0.0003	-2.0486	0.0405	0.9916
Part of a multiple birth	1777	-0.0030	0.0015	-2.0483	0.0405	0.9916

© 2023 Koller D et al. *JAMA Network Open.*

Activities undertaken to treat depression: Other therapeutic activities such as mindfulness, yoga or art classes	20547 3	-0.0107	0.0052	-2.0390	0.0415	0.9916
Whole body water mass	23102 irnt	-0.0198	0.0097	-2.0318	0.0422	0.9916
Diagnoses - main ICD10: C44 Other malignant neoplasms of skin	C44	-0.0030	0.0015	-2.0290	0.0425	0.9916
Treatment/medication code: mirena 20mcg/24hrs intrauterine system	20003 1140921822	-0.0011	0.0005	-2.0251	0.0429	0.9916
Diagnoses - main ICD10: J98 Other respiratory disorders	J98	0.0008	0.0004	2.0246	0.0429	0.9916
Behavioural and miscellaneous addictions: A behaviour	20552 2	0.0026	0.0013	2.0198	0.0434	0.9916
Diagnoses - main ICD10: O47 False labour	O47	-0.0013	0.0006	-2.0183	0.0436	0.9916
Trunk predicted mass	23130 irnt	-0.0196	0.0097	-2.0165	0.0437	0.9916
Diagnoses - main ICD10: J44 Other chronic obstructive pulmonary disease	J44	0.0013	0.0006	2.0087	0.0446	0.9916
Trunk fat-free mass	23129 irnt	-0.0195	0.0097	-2.0058	0.0449	0.9916
Treatment/medication code: allopurinol	20003 1140875408	0.0008	0.0004	2.0041	0.0451	0.9916
Non-cancer illness code, self-reported: spinal injury	20002 1267	0.0007	0.0003	1.9959	0.0459	0.9916
3mm asymmetry index (left)	5156 irnt	0.0436	0.0218	1.9959	0.0460	0.9916
Cereal type: Biscuit cereal (e.g. Weetabix)	1468 2	0.0079	0.0040	1.9922	0.0464	0.9916
Nitrogen dioxide air pollution; 2006	24017 irnt	0.0195	0.0098	1.9912	0.0465	0.9916
Particulate matter air pollution (pm10); 2007	24019 irnt	0.0188	0.0094	1.9906	0.0465	0.9916
Non-cancer illness code, self-reported: clotting disorder/excessive bleeding	20002 1445	-0.0007	0.0003	-1.9903	0.0466	0.9916
Treatment/medication code: sotalol	20003 1140879854	0.0007	0.0003	1.9897	0.0466	0.9916

© 2023 Koller D et al. *JAMA Network Open.*

Other specified/unspecified disorders of synovium and tendon +Other specified/unspecified bursopathies	M13 DISSYNOTENDNAS	0.0006	0.0003	1.9896	0.0466	0.9916
Job coding: personnel or industrial relations officer, recruitment consultant	22601 35623213	0.0029	0.0015	1.9883	0.0468	0.9916
Diagnoses - main ICD10: M80 Osteoporosis with pathological fracture	M80	0.0005	0.0003	1.9877	0.0468	0.9916
Treatment/medication code: food supplement/plant/herbal extract	20003 1199	-0.0014	0.0007	-1.9867	0.0470	0.9916
Treatment/medication code: meloxicam	20003 1140926732	-0.0012	0.0006	-1.9853	0.0471	0.9916
Reason for reducing amount of alcohol drunk: Doctor's advice	2664 2	0.0032	0.0016	1.9829	0.0474	0.9916
Actions taken following self-harm: See anyone from psychiatric or mental health services, including liaison services	20554 1	0.0044	0.0022	1.9774	0.0480	0.9916
Basal metabolic rate	23105 irnt	-0.0193	0.0097	-1.9769	0.0481	0.9916
Non-cancer illness code, self-reported: unclassifiable	20002 99999	0.0039	0.0020	1.9720	0.0486	0.9916
Arm predicted mass (left)	23126 irnt	-0.0195	0.0099	-1.9719	0.0486	0.9916
Gap coding: Unable to work due to sickness or disability	22660 106	-0.0075	0.0038	-1.9705	0.0488	0.9916
Job coding: library assistant/clerk, press cuttings clerk	22601 41353316	-0.0041	0.0021	-1.9704	0.0488	0.9916
Type milk consumed: did not have milk	100920 0	0.0117	0.0059	1.9651	0.0494	0.9916
6mm asymmetry angle (right)	5109 irnt	-0.0548	0.0279	-1.9635	0.0496	0.9916

© 2023 Koller D et al. *JAMA Network Open.*

eTable 12. Association of the rs1266606 Variant With Phenotypic Traits Available From the GWAS Atlas

Atlas ID	PMID	Year	Domain	Trait	P-value	N	EA	NEA
3271	31427789	2019	Skeletal	Comparative height size at age 10	0.000396	380167	G	A
3187	31427789	2019	Skeletal	Standing height	0.000709	385748	G	A
657	24816252	2014	Metabolic	::::X-03094	0.00079	7804	A	G
600	24816252	2014	Metabolic	Peptide::Fibrinogen cleavage peptide::DSGEGDFXAEGGGVR*	0.00137	5371	A	G
674	24816252	2014	Metabolic	::::X-08402	0.001833	7726	A	G
4660	BioRxiv: https://doi.org/10.1101/288568	2019	Neurological	Posterior limb of internal capsule fractional anisotropy	0.001911	17706	G	A
4746	BioRxiv: https://doi.org/10.1101/288654	2019	Neurological	Inferior fronto-occipital fasciculus radial diusivities	0.002372	17706	A	G
138	23754948	2013	Metabolic	Waist-hip ratio (male)	0.0039	34629	A	G
4198	31217584	2019	Metabolic	Waist-hip ratio (male)	0.004188	24838	G	A
3362	31427789	2019	Mortality	Mother's age at death	0.004318	227076	G	A
3250	31427789	2019	Nutritional	Pork intake	0.004653	384328	A	G
599	24816252	2014	Metabolic	Peptide::Fibrinogen cleavage peptide::ADSGEGDFXAEGGGVR*	0.004728	5588	A	G
472	24816252	2014	Metabolic	Lipid::Bile acid metabolism::taurodeoxycholate	0.004803	1564	G	A

© 2023 Koller D et al. *JAMA Network Open.*

4748	BioRxiv: https://doi.org/10.1101/288656	2019	Neurological	Posterior limb of internal capsule radial diusivities	0.005027	17706	A	G
4664	BioRxiv: https://doi.org/10.1101/288572	2019	Neurological	Superior corona radiata fractional anisotropy	0.005161	17706	G	A
4268	29892015	2018	Cardiovascular	Atrial Fibrillation	0.005501	588190	G	A
181	25673412	2015	Metabolic	Waist-hip ratio (male)	0.0065	94434	A	G
182	25673412	2015	Metabolic	Waist-hip ratio (male)	0.0065	100032	A	G
4659	BioRxiv: https://doi.org/10.1101/288567	2019	Neurological	Posterior corona radiata fractional anisotropy	0.006718	17706	G	A
791	24816252	2014	Metabolic	::::X-12704	0.007125	1813	A	G
132	23754948	2013	Metabolic	Waist circumference (male)	0.0074	36231	A	G
187	25673412	2015	Metabolic	Waist-hip ratio (male, adjusted for BMI)	0.0074	93480	A	G
188	25673412	2015	Metabolic	Waist-hip ratio (male, adjusted for BMI)	0.0074	99078	A	G
3874	27863252	2016	Immunological	Eosinophil count (three-way meta)	0.007416	172275	G	A
4662	BioRxiv: https://doi.org/10.1101/288570	2019	Neurological	Retrolenticular part of internal capsule fractional anisotropy	0.007967	17706	G	A
3939	28892062	2017	Metabolic	Body Mass Index	0.008162	158284	A	G
1011	27989323	2017	Immunological	Tumor necrosis factor beta	0.008825	1559	A	G
4355	30206230	2018	Immunological	IL-1beta in gingival crevicular	0.009077	4910	G	A

© 2023 Koller D et al. *JAMA Network Open.*

948	27005778	2016	Metabolic	Total fatty acids	0.009187	13505	A	G
4740	BioRxiv: https://doi.org/10.1101/288648	2019	Neurological	Cingulum (hippocampus) radial diusivities	0.009559	17706	A	G
4658	BioRxiv: https://doi.org/10.1101/288566	2019	Neurological	Inferior fronto-occipital fasciculus fractional anisotropy	0.009567	17706	G	A
747	24816252	2014	Metabolic	::::X-12029	0.01031	7564	G	A
675	24816252	2014	Metabolic	::::X-08766	0.01049	6050	A	G
4122	29403010	2018	Metabolic	Gamma-glutamyl transferase	0.01083	118309	G	A
4702	BioRxiv: https://doi.org/10.1101/288610	2019	Neurological	Inferior fronto-occipital fasciculus mean diusivities	0.01134	17706	A	G
205	21355061	2011	Metabolic	Microalbuminuria	0.012	30482	G	A
3252	31427789	2019	Nutritional	Bread intake	0.01265	377627	G	A
949	27005778	2016	Metabolic	Total phosphoglycerides	0.012957	13519	A	G
3674	31427789	2019	Gastrointestinal	Diagnoses - main ICD10: K62 Other diseases of anus and rectum	0.01328	300791	G	A
869	27005778	2016	Metabolic	OmegaL7 and L9 and saturated fatty acids	0.013501	13506	A	G
3929	28749367	2017	Cardiovascular	QT interval	0.01402	22158	G	A
369	24816252	2014	Metabolic	Amino acid::Creatine metabolism::creatine	0.01409	7822	A	G
930	27005778	2016	Metabolic	Phosphatidylcholine and other cholines	0.0143	13542	A	G
3254	31427789	2019	Nutritional	Salt added to food	0.01475	386322	A	G

© 2023 Koller D et al. *JAMA Network Open.*

848	24816252	2014	Metabolic	Xenobiotics::Food component/Plant::X-14977--vanillin	0.01499	1789	A	G
4649	BioRxiv: https://doi.org/10.1101/288557	2019	Neurological	Average across all tracts fractional anisotropy	0.01616	17706	G	A
4098	29403010	2018	Metabolic	Total cholesterol	0.01718	128305	G	A
572	24816252	2014	Metabolic	Lipid::Sterol, Steroid::androsterone sulfate	0.01756	7785	G	A
4750	BioRxiv: https://doi.org/10.1101/288658	2019	Neurological	Retrolenticular part of internal capsule radial diusivities	0.01766	17706	A	G
569	24816252	2014	Metabolic	Lipid::Sterol, Steroid::4-androsten-3beta,17beta-diol disulfate 2*	0.01782	7776	G	A
3555	31427789	2019	Activities	Medication for cholesterol, blood pressure, diabetes, or take exogenous hormones: Blood pressure medication	0.01863	207533	A	G
3407	31427789	2019	Social Interactions	Number of older siblings	0.01892	111284	A	G
3665	31427789	2019	Neoplasms	Diagnoses - main ICD10: C44 Other and unspecified malignant neoplasm of skin	0.01973	300791	G	A
4667	BioRxiv: https://doi.org/10.1101/288575	2019	Neurological	Sagittal stratum fractional anisotropy	0.01993	17706	G	A

© 2023 Koller D et al. *JAMA Network Open.*

577	24816252	2014	Metabolic	Lipid::Sterol, Steroid::epiandrosterone sulfate	0.02001	7769	G	A
3873	27863252	2016	Immunological	Sum eosinophil basophil count (three-way meta)	0.02029	171771	G	A
3641	31427789	2019	Environment	Illnesses of mother: Bowel cancer	0.02043	367939	A	G
598	24816252	2014	Metabolic	Peptide::Fibrinogen cleavage peptide::ADpSGEGDFXAEGGGVR*	0.02049	3939	A	G
4654	BioRxiv: https://doi.org/10.1101/288562	2019	Neurological	External capsule fractional anisotropy	0.02164	17706	G	A
185	25673412	2015	Metabolic	Waist-hip ratio (adjusted for BMI)	0.022	210086	A	G
186	25673412	2015	Metabolic	Waist-hip ratio (adjusted for BMI)	0.022	224456	A	G
4256	30952852	2019	Psychiatric	Sleep midpoint	0.022	84810	A	G
4373	30367059	2018	Endocrine	Free thyroxine (FT4, male)	0.02255	22455	A	G
3296	31427789	2019	Psychiatric	Risk taking	0.02301	372651	A	G
3941	28892062	2017	Metabolic	Body Mass Index (female)	0.02305	72390	A	G
4652	BioRxiv: https://doi.org/10.1101/288560	2019	Neurological	Cingulum (hippocampus) fractional anisotropy	0.02305	17706	G	A
4755	BioRxiv: https://doi.org/10.1101/288663	2019	Neurological	Sagittal stratum radial diusivities	0.02337	17706	A	G

© 2023 Koller D et al. *JAMA Network Open.*

3660	31427789	2019	Neoplasms	Cancer register - Histology of cancer tumour: Basal cell carcinoma, NOS	0.02383	60692	G	A
3838	27863252	2016	Immunological	Eosinophil count (two-way meta)	0.023842	131999	G	A
3383	31427789	2019	Cognitive	Prospective memory test - Number of attempts	0.02404	128912	A	G
3691	31427789	2019	Cardiovascular	Diagnoses - secondary ICD10: I10 Essential (primary) hypertension	0.02433	244890	A	G
4696	BioRxiv: https://doi.org/10.1101/288604	2019	Neurological	Cingulum (hippocampus) mean diusivities	0.02464	17706	A	G
3698	31427789	2019	Gastrointestinal	Diagnoses - secondary ICD10: K44 Diaphragmatic hernia	0.025	244890	A	G
378	24816252	2014	Metabolic	Amino acid::Glutathione metabolism::cysteine-glutathione disulfide	0.02517	1997	G	A
3595	31427789	2019	Activities	Mineral and other dietary supplements: Zinc	0.02576	385261	G	A
868	27005778	2016	Metabolic	OmegaL6 fatty acids	0.026077	13506	A	G
4726	BioRxiv: https://doi.org/10.1101/288634	2019	Neurological	Posterior limb of internal capsule mode of anisotropy	0.02661	17706	G	A
3401	31427789	2019	Ear, Nose, Throat	Loud music exposure frequency	0.02673	127474	A	G
4538	31676860	2019	Neurological	Right hippocampus	0.02715	19629	G	A
739	24816252	2014	Metabolic	::::X-11850	0.02809	4868	G	A
4155	29403010	2018	Cardiovascular	E/A ratio	0.02836	8600	G	A

528	24816252	2014	Metabolic	Lipid::Long chain fatty acid::stearate (18:0)	0.02864	7803	G	A
3270	31427789	2019	Metabolic	Comparative body size at age 10	0.02981	379749	G	A
179	25673412	2015	Metabolic	Waist-hip ratio	0.03	212248	A	G
180	25673412	2015	Metabolic	Waist-hip ratio	0.03	226643	A	G
4675	BioRxiv: https://doi.org/10.1101/288583	2019	Neurological	Corticospinal tract axial diusivities	0.03028	17706	A	G
767	24816252	2014	Metabolic	::::X-12236	0.03105	1924	A	G
3968	29273806	2018	Respiratory	Asthma (fixed effect model)	0.031824	127669	G	A
462	24816252	2014	Metabolic	Energy::Oxidative phosphorylation::acetylphosphate	0.03281	7789	A	G
4043	30124842	2018	Skeletal	Height	0.033	693529	G	A
4525	31676860	2019	Neurological	Left hippocampus	0.03358	19629	G	A
724	24816252	2014	Metabolic	::::X-11552	0.0342	2170	G	A
648	24816252	2014	Metabolic	Xenobiotics::Xanthine metabolism::theobromine	0.03432	7768	G	A
3589	31427789	2019	Activities	Types of physical activity in last 4 weeks: Heavy DIY (eg: weeding, lawn mowing, carpentry, digging)	0.0345	384450	G	A
742	24816252	2014	Metabolic	::::X-11859	0.03456	2721	A	G
814	24816252	2014	Metabolic	::::X-13069	0.03484	6770	G	A
4259	30946739	2019	Activities	Facial attractiveness (female-coder, male samples)	0.03541	1792	G	A
23	26176920	2015	Psychiatric	Major depressive disorder (females)	0.03544	10640	A	G

© 2023 Koller D et al. *JAMA Network Open.*

1117	27918534	2017	Metabolic	Pericardial adipose tissue volume	0.036	12204	NA	NA
3292	31427789	2019	Psychiatric	Worry too long after embarrassment	0.03634	370660	G	A
3598	31427789	2019	Cardiovascular	Non-cancer illness code, self-reported: angina	0.03706	289307	A	G
4318	30649302	2019	Reproduction	Anti-Mullerian hormone	0.0375	3344	G	A
4509	31676860	2019	Neurological	Cerebellar vermal lobules VIII X	0.03758	19629	G	A
4375	30367059	2018	Endocrine	Hyperthyroidism	0.03771	51823	A	G
66	27416945	2016	Endocrine	Insulin sensitivity index (combined influence of the genotype effect adjusted for BMI and the interaction effect between the genotype and BMI on ISI)	0.03775	16753	A	G
3419	31427789	2019	Cognitive	Pairs matching test - Number of correct matches in round	0.03777	95708	G	A
3999	29500382	2018	Psychiatric	Worry too long after embarrassment (WORR-EMB)	0.03824	261094	G	A
606	24816252	2014	Metabolic	Peptide::gamma-glutamyl::gamma-glutamylphenylalanine	0.03841	7753	G	A
619	24816252	2014	Metabolic	Xenobiotics::Chemical::glycerol 2-phosphate	0.0388	5912	G	A
3875	27863252	2016	Immunological	Eosinophil percentage of white cells (three-way meta)	0.03964	172378	G	A
665	24816252	2014	Metabolic	::::X-05907	0.04014	7734	A	G
3263	31427789	2019	Psychiatric	Average weekly champagne plus white wine intake	0.04106	273869	A	G
4156	30531953	2018	Neurological	Epilepsy	0.04133	44889	G	A

© 2023 Koller D et al. *JAMA Network Open.*

4737	BioRxiv: https://doi.org/10.1101/288645	2019	Neurological	Average across all tracts radial diusivities	0.04336	17706	A	G
3486	31427789	2019	Nutritional	Bread type: Brown	0.04338	372617	A	G
4711	BioRxiv: https://doi.org/10.1101/288619	2019	Neurological	Sagittal stratum mean diusivities	0.04342	17706	A	G
4742	BioRxiv: https://doi.org/10.1101/288650	2019	Neurological	External capsule radial diusivities	0.04356	17706	A	G
4338	30531941	2018	Psychiatric	Sleep durataion (conditioning sex and BMI)	0.044	91105	G	A
4639	31676860	2019	Neurological	Right hippocampus	0.04406	21821	G	A
3437	31427789	2019	Respiratory	Doctor diagnosed hayfever or allergic rhinitis	0.04492	97640	G	A
25	24839885	2014	Psychiatric	Internalizing	0.0452	4596	G	A
697	24816252	2014	Metabolic	::::X-11381	0.04552	7753	A	G
928	27005778	2016	Metabolic	MonoLunsaturated fatty acids	0.045637	13535	A	G
3916	28073927	2017	Ophthalmological	Disc area	0.04684	7307	G	A
3839	27863252	2016	Immunological	Eosinophil percentage of white cells (two-way meta)	0.047265	132052	G	A
3969	29273806	2018	Respiratory	Asthma (random effect model)	0.048403	127669	G	A
3308	31427789	2019	Mortality	Long-standing illness, disability or infirmity	0.04845	377498	G	A

© 2023 Koller D et al. *JAMA Network Open.*

689	24816252	2014	Metabolic	::::X-11247	0.04854	7403	A	G
4610	31676860	2019	Neurological	Cerebellar vermal lobules VIII X	0.04904	21821	G	A
645	24816252	2014	Metabolic	Xenobiotics::Xanthine metabolism::7-methylxanthine	0.04928	5901	G	A
608	24816252	2014	Metabolic	Peptide::gamma-glutamyl::gamma-glutamyltyrosine	0.0493	7468	G	A
682	24816252	2014	Metabolic	::::X-10395	0.04999	7784	A	G

© 2023 Koller D et al. *JAMA Network Open.*
