# Supplementary material for: Epidemiologic and Genetic Associations of Endometriosis With Depression, Anxiety, and Eating Disorders
Source: JAMA Netw Open. 2023 Jan 18;6(1):e2251214. doi: 10.1001/jamanetworkopen.2022.51214 (PMC9856929; doi:10.1001/jamanetworkopen.2022.51214)
Supplement: Supplement 2. — Data Sharing Statement [file jamanetwopen-e2251214-s002.pdf]

Data Sharing Statement

Koller. Epidemiologic and Genetic Associations of Endometriosis With Depression, Anxiety, and Eating Disorders. *JAMA Netw Open*. Published January 18, 2023. doi:10.1001/jamanetworkopen.2022.51214

Data
Data available: No

Additional Information
Explanation for why data not available: The data used in this study are already publicly available. Individual-level data are available via application to UK Biobank. The other datasets are available in the following websites: MVP depression and anxiety GWAS summary statistics: https://www.ncbi.nlm.nih.gov/projects/gap/cgi-bin/study.cgi?study_id=phs001672.v7.p1 PGC anorexia nervosa GWAS summary statistics: https://www.med.unc.edu/pgc/download-results/ FinnGen endometriosis GWAS summary statistics: https://r6.finngen.fi/
